# Access Path to the Ligand Binding Pocket May Play a Role in Xenobiotics Selection by AhR

**DOI:** 10.1371/journal.pone.0146066

**Published:** 2016-01-04

**Authors:** Dániel Szöllősi, Áron Erdei, Gergely Gyimesi, Csaba Magyar, Tamás Hegedűs

**Affiliations:** 1 MTA-SE Molecular Biophysics Research Group, Hungarian Academy of Sciences, Budapest, 1094, Hungary; 2 Department of Biophysics and Radiation Biology, Semmelweis University, Budapest, 1094, Hungary; 3 Faculty of Information Technology and Bionics, Pázmány Péter Catholic University, Budapest, 1083, Hungary; 4 Institute of Biochemistry and Molecular Medicine, University of Bern, Bern, 3012, Switzerland; 5 Institute of Enzymology, Research Centre for Natural Sciences, Hungarian Academy of Sciences, Budapest, 1117, Hungary; University of Copenhagen, DENMARK

## Abstract

Understanding of multidrug binding at the atomic level would facilitate drug design and strategies to modulate drug metabolism, including drug transport, oxidation, and conjugation. Therefore we explored the mechanism of promiscuous binding of small molecules by studying the ligand binding domain, the PAS-B domain of the aryl hydrocarbon receptor (AhR). Because of the low sequence identities of PAS domains to be used for homology modeling, structural features of the widely employed HIF-2α and a more recent suitable template, CLOCK were compared. These structures were used to build AhR PAS-B homology models. We performed molecular dynamics simulations to characterize dynamic properties of the PAS-B domain and the generated conformational ensembles were employed in *in silico* docking. In order to understand structural and ligand binding features we compared the stability and dynamics of the promiscuous AhR PAS-B to other PAS domains exhibiting specific interactions or no ligand binding function. Our exhaustive *in silico* binding studies, in which we dock a wide spectrum of ligand molecules to the conformational ensembles, suggest that ligand specificity and selection may be determined not only by the PAS-B domain itself, but also by other parts of AhR and its protein interacting partners. We propose that ligand binding pocket and access channels leading to the pocket play equally important roles in discrimination of endogenous molecules and xenobiotics.

## Introduction

Resistance to chemotherapy is the major cause of the unsuccessful treatment of cancers. Major players in multidrug resistance are ABC (ATP Binding Cassette) membrane transporters, which are located in the plasma membrane and pump out xenobiotics from the cell in an ATP dependent manner [1,2]. Although high resolution structures of full length ABC proteins have been determined in different conformations, the mechanism of drug binding and transport is largely unknown [3]. Details of the recognition of chemically unrelated compounds at the atomic level may help in the rational design of small molecules either to evade or inhibit these proteins. However, the large size and hydrophobic nature of ABC transporters bring significant difficulties in characterizing their multidrug binding properties employing either experimental or computational approaches.

It is very important to realize that there are other cellular proteins, which also recognize xenobiotics, including drug metabolizing enzymes and multi-ligand binding transcription factors. These soluble proteins together with membrane transporters act in a network forming the chemoimmune system to protect the cell from harmful molecules [1]. Phase I (e.g. oxidation by cytochrome P450s, CYPs) and phase II (e.g. conjugation by glutathione S-transferases) metabolic enzymes in the cell convert xenobiotics to a less toxic product [4,5]. Some ABC transporters take part in both limiting the entry of xenobiotics into the cell (phase 0) and extruding their metabolized forms (phase III) [2,6]. All these processes influence the ADME-Tox (Absorption, Distribution, Metabolism, Excretion, and Toxicity) properties of drugs and result in a concentration of drugs below the effective intracellular level, preventing them to act on their targets.

Although there are many structural studies aiming to explain the basis of promiscuous binding of nuclear receptors and metabolic enzymes, the understanding of multidrug recognition and the generation of predictive models for *in silico* screening of substrates and ligands are still challenging [7–9].

**Fig 1:**
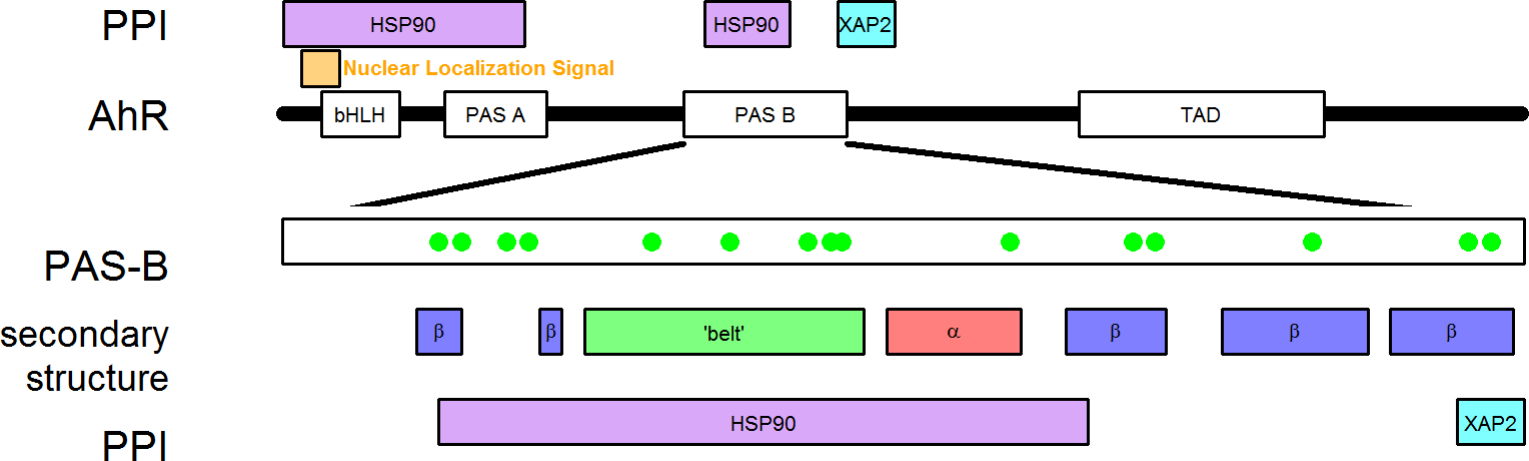
The ligand binding competent AhR is part of a cytoplasmic protein complex. In the cytoplasmic AhR complex Hsp90 has been indicated to bind AhR at two locations. At the N-terminal region it overlaps with the nuclear localization signal (orange box), bHLH, and PAS A domain. Hsp90 also interacts with the PAS B domain, while XAP2 binds C-terminal of this AhR domain. Secondary structure elements of the PAS-B domain (α/β) and the belt region are indicated. AhR interactions with Hsp90 and XAP2 are labeled with magenta and turquoise boxes, respectively. Green dots indicate TCDD binding amino acids. PPI: protein-protein interactions.

In order to investigate the details of the interaction of proteins with multiple small molecules including xenobiotics and drugs, we selected the small soluble promiscuous ligand binding C-terminal PAS (or PAS-B) domain of the human aryl hydrocarbon receptor (AhR). PAS stands for Per-Arnt-Sim domains from Period circadian protein, Aryl hydrocarbon receptor nuclear translocator protein, and Single-minded protein. AhR is a ligand-dependent transcription factor regulating a broad spectrum of biological processes including detoxification, development, cellular oxidation/antioxidation, responding to ultraviolet light, melanogenesis, inflammation, and regulation of immune signaling [10,11]. Functional domains of AhR are the basic helix-loop-helix domain (bHLH) responsible for DNA binding, two PAS domains of which the C-terminal PAS-B is the ligand binding domain, and a transactivation domain (TAD) at the far C-terminus [10,12] (Fig 1). AhR can be found in the cytoplasm in complex with two Hsp90 (heat shock protein 90) molecules, a XAP2 protein (hepatitis B virus X-associated protein 2), and the p23 co-chaperone (Hsp23) [13]. p23 interacts only with Hsp90 and stabilizes the conformation of the chaperone in the ATP-bound conformation [10,14], which has been suggested to be more rigid compared to the apo conformation [15]. The potential functions of XAP2 are contradictory but might involve modulating the localization of the AhR complex and its sensitivity to ligand binding [16]. A large part of AhR, including the bHLH and both PAS domains, has been reported to interact with the Hsp90 homodimer. It has been suggested that one Hsp90 molecule is bound only within the PAS region while the other Hsp90 appeared to require interaction both with the bHLH and also with the PAS regions [10,17]. The bHLH domain of AhR also contains a nuclear localization signal (NLS) [18] which signals the transport of the complex into the nucleus upon ligand binding [18], where AhR dissociates and dimerizes with the Aryl hydrocarbon Receptor Nuclear Translocator (ARNT). This nuclear complex is capable of binding to xenobiotic response elements (XRE), interacting with transcriptional activators and repressors, thus influencing the transcription from the corresponding promoters [19,20].

AhR PAS-B domain is a relatively small domain consisting of approximately 110 amino acids which can bind various endogenic and xenobiotic compounds from which the most prototypical and studied is 2,3,7,8-tetrachlorodibenzo-p-dioxin (TCDD, dioxin) [20]. Although the structure of the PAS-B domain has not been determined so far, the high structural similarity of PAS domains allows to build reliable homology models in spite of their low sequence similarity [21,22]. These models have been based on PAS domain structures of HIF-2α (PDBIDs: 3F1P, 3H82 [7]; 1P97, 1X0O [23,24]; 3F1O, 3H7W, 3H82 [25]; and 1P97 [26]) and multiple PAS structures have also been employed simultaneously as templates (PDBIDs: 1P97, 1X0O, 1WA9, 1BYW, 1LL8, 1G28, 1OJ5, 1DRM, 1NWZ) [27]. Many past studies have used homology models to investigate the interaction between docked ligands and specific amino acids putatively involved in ligand binding, in many cases complemented with mutational experiments [7,23–27]. Some of these investigations have used a limited number of ligands, which were usually AhR agonists with high affinity, for *in silico* docking simulations. Moreover, most calculations have been carried out on one energy minimized conformation without taking the inherent flexibility of the PAS-B domain into account. Motto *et al*. employed a more advanced approach and validated their structural model of rat AhR ligand binding domain by docking 14 polychlorodibenzo-p-dioxins to a conformational ensemble generated via clustering of homology models [25]. However, the selection and energy minimization of the homology models were performed in the presence of a ligand inside the putative binding cavity of the model, thus the resulting structures might have been biased to form a specific conformation for accommodating a given ligand. Nuti *et al*. [28] have built homology models based on HIF-2α structures containing a bound ligand and performed MD simulations using complexes with docked TCDD or endogenous AhR ligands [28]. Their results suggested that different ligands interact with different amino acids and change the conformational landscape of the PAS-B domain differently. Unfortunately, differences between conformations and data on dynamics other than RMSD values have not been presented.

The aim of our study was to gain insight into the multidrug recognition by analyzing the dynamics and the ligand binding properties of the AhR PAS-B domain. Since flexibility has been suggested to be important in recognizing multiple molecules, we generated conformational ensembles employing different types of molecular dynamic simulation techniques using homology models based on different template structures including the widely used HIF-2α and also CLOCK with higher sequence similarity. The ensembles were used for the docking of various ligands ranging from high affinity ligands to compounds not interacting with the AhR PAS-B domain. The similar binding pattern of drugs with highly different affinities suggests that the ligand recognition also involves other entities than the PAS-B domain, such as other parts of AhR and protein interaction partners in the cytosolic complex. We suggest that access pathways to binding pockets may play as important roles in the ligand or substrate selection in the case of AhR and ABC multidrug transporters as indicated for CYP450s and nuclear receptors [29–31]. Therefore this composite recognition process may provide a general mechanism for ligand and substrate selections for proteins with highly promiscuous and hydrophobic binding pocket.

## Materials and Methods

### Structural models

Two homology models of the human AhR PAS-B domain (amino acids 283–392) were built, one based on the HIF-2α (PDBID:1P97; [32]) and the other based on the more recent CLOCK protein PAS-B domain in the CLOCK/BMAL dimer (PDBID:4F3L; [33]). The latter one exhibits somewhat higher sequence similarity to AhR PAS-B (BLAST identity: 28%, E-value: 2.64*10^−9^), while the first template has been used in many earlier studies (BLAST identity: 27%, E-value: 9.40*10^−7^). 1P97 is a NMR structural ensemble containing 20 structures. The template was selected using OLDERADO as in [27].

The alignments, which were created with ClustalW 2.1 [34] and used as input for Modeller 9.12 [35], are shown in S1 Fig. We generated 100 homology models and selected the model based on the lowest DOPE (Discrete Optimized Protein Energy) score [36]. The selected models were further optimized by the energy minimization protocol of Chiron [37,38]. The quality of the optimized models was checked by various methods. The z-score of ProSA [39] and QMEAN [40] were calculated for each model to assess the overall quality of the structures. ProCheck [41] was employed to gain detailed information on the stereochemistry of the structural models. This method lists all residues in favored, allowed, and disallowed regions of the Ramachandran plot.

Other PAS containing protein structures were also analyzed to study PAS domain dynamics, their detailed properties (e.g. names, PDB IDs) are presented in Table 1.

**Table 1:** PAS containing structures used in DMD equilibrium simulations.

Name	UniProt entry name	PDBID	Ligand binding
Sensor protein FixL [40]	FIXL_RHIME	1EW0	YES^*^^#^
Nitrogen fixation regulatory protein [43]	NIFL_AZOVI	2GJ3	YES
Aryl hydrocarbon receptor nuclear translocator-like protein 2	BMAL2_HUMAN	2KDK	NO
Blue-light photoreceptor, Sensor protein fixL [48]	PHOT_BACSU	2PR5	YES
Sensor protein DCUS [64]	DCUS_ECOLI	2W0N	YES
Phototropin-1 [65]	PHOT1_ARATH	2Z6C	YES^*^
Aryl hydrocarbon nuclear translocator [73,74]	ARNT_HUMAN	3F1P_B	NO
Period circadian protein homolog 2 [75]	PER2_MOUSE	3GDI	NO
Drosophila PERIOD	PER_DROME	3GEC	NO
Erythrobacter litoralis El222 [76]	Q2NB98_ERYLH	3P7N	YES
Potassium voltage-gated channel subfamily H member 1 [77]	KCNH1_MOUSE	4HOI	NO
Potassium voltage-gated channel subfamily H member 2 [77]	KCNH2_HUMAN	4HQA	NO^#^
Endothelial PAS domain-containing protein 1 [73,74]	EPAS1_HUMAN	3F1P_A	YES^#^
Photoactive yellow protein [78]	PYP_HALHA	1NWZ	YES^*^^#^

*contains prosthetic group

^#^employed in replica exchange simulation

In order to analyze the intradomain residue-residue interactions in the AhR homology models and their template structures we employed the MDAnalysis toolkit and its elastic network analysis module [42,43]. The energy minimized homology models and template structures were used as input and a pair of amino acids was considered connected when their Cα atoms were closer than 7Å, the default value in the module. To allow the visual examination of the connected residues, we created a network arrangement resembling the cartoon representation of the PAS domain [27].

### Molecular dynamics simulations

Equilibrium simulations were carried out using GROMACS 4.6.1 [44–47] with two different force fields. We employed CHARMM36 [48] and also GROMOS43a2, as it has been used before for AhR PAS-B homology model simulations in a similar study [27]. The structures were solvated in water (SPC for GROMOS and TIP3P for CHARMM) in a cubic box with periodic boundary conditions. The dimensions of the box were set to allow at least 0.8 nm between protein and box walls on each side. After addition of ions to neutralize the system, a short minimization with the steepest descent method was performed until the maximal force in the system was lower than 10 kJ/mol/nm in the case of CHARMM and 1,000 kJ/mol/nm in the case of GROMOS. For CHARMM36 simulations a two-step equilibration was performed, a constant volume and temperature (NVT, T = 300 K) setup was followed by one with constant pressure and temperature (NPT, p = 1 bar, T = 300 K). Both equilibration steps were 100 ps long. For GROMOS simulations the energy minimized structures were equilibrated for 1 ns while the pressure and temperature were kept constant (NPT, p = 1 bar, T = 300 K). The potential energy change was checked at each step for all the systems and indicated sufficient level of minimization (not shown). The equilibrated systems were used as starting points for simulations. All production simulations (n = 3 for each system) were carried out for 50 ns and coordinates were saved at every 20 ps resulting in 2,500 structures for each simulation. For long-range electrostatic interactions, the Particle Mesh Ewald summation method was employed. Van der Waals interactions were described by a 6–12 Lennard-Jones potential with a distance cutoff of 1.0 nm and 0.9 nm for simulations with CHARMM and GROMOS, respectively. For short-range electrostatic cutoff the same values were used. Protein and solvent were independently coupled to a thermal bath by velocity rescaling (CHARMM) and a Berendsen thermostat (GROMOS) at 300 K and a coupling coefficient of 0.1 ps. For pressure coupling the Parrinello-Rahman barostat was used at 1 bar with a coupling coefficient of 2 ps. The internal degrees of freedom of water molecules and all bond distances in the proteins were constrained by the LINCS algorithm.

In order to have more divergent conformations from the starting structure and also to be able performing replica exchange simulations on reasonable time scales, all-atom discrete molecular dynamics (DMD) was employed [37,49,50]. DMD is an event (atomic collision) driven simulation method using a discrete potential energy function that reduces the amount of calculations, as there is no need to compute forces and accelerations and causes an adaptive time step. Because of the discretized nature of DMD simulations, internal time and temperature units are used instead of regular units. Given the units of mass [M, dalton, 1.66×10^−24^ gram], length [L, angstrom, 10^−10^ m], and energy [E, kcal/mol], the time unit can be calculated as [L]·([M]/[E])^0.5^, which is approximately 50 femtosecond. The temperature unit is kcal/mol·k_B_ or 5.03×10^2^ Kelvin, where k_B_ is the Boltzmann constant [49]. Equilibrium DMD simulations were done at 0.53 and 0.59 temperature units (approx. 267K and 297K, respectively) for 10^6^ time units, which is approximately 50 ns of real time and coordinates were saved at every 200 time units resulting in 5,000 structures for every simulation. Simulations with two different temperature setups were performed to enhance the conformational sampling and ensure that relevant structures are generated for further analysis. For temperature coupling, Andersen's thermostat was used and the heat exchange factor was set to 0.1. Three parallel simulations were started for each structure and temperature. Replica exchange (RX) simulations were also performed for 10^6^ time units at 0.5246, 0.5451, 0.5665, 0.5886, 0.6116, 0.6355, 0.6604 and 0.6862 temperature units with the same settings as in the equilibrium simulations. Temperatures were selected to ensure at least 25% exchange probability between neighboring temperatures. Conditions for replica exchange were tested every 1,000 time units and frames were saved every 200 time units, thus 5,000 conformations were generated for each replica. To analyze the thermostability, the molar heat capacity as a function of temperature was calculated from the RX simulations using the Weighted Histogram Analysis Method (WHAM) [51] (http://www.hegelab.org/pywham.py). The πDMD software employed for DMD simulations was kindly provided by Molecules in Action, LLC (http://www.moleculesinaction.com).

### Analysis of the conformational ensembles

GROMACS tools were used to evaluate the flexibility of the simulated models by calculating root mean squared deviation (RMSD, calculated with g_rms) and root mean squared fluctuations (RMSF, calculated with g_rmsf) for the C_α_ atoms, with the starting structure used as the reference. While RMSD values indicate the overall flexibility of the whole structure during a simulation, RMSF values show the flexibility of a protein at the amino acid level averaged for the whole simulation. Results of the parallel simulations were averaged and plotted with ± standard deviation indicated as a similarly colored band around the RMSD or RMSF curve.

Potential ligand binding pockets were analyzed using MDpocket, which is included in Fpocket 2.0 [52,53], with default parameters. The software searches for internal pockets in an ensemble based on a predefined structure, which was defined as the energy minimized starting structure. The grid cut-off was set to 0.5, which enabled us to find small and/or transient pockets.

### Molecular docking

In order to take the dynamic properties of PAS domains into account in ligand binding, we performed docking to the conformational ensembles generated by equilibrium simulations. To complete this in a rational time frame, AutoDock Vina 1.1.2 [54] was employed. Preparation of the structures was done with python scripts delivered by AutoDockTools. The search space for docking contained the whole structure, thus the ligand could bind to any part of the protein (S2 Fig). Docking exhaustiveness and number of models were set to 64 and 100, respectively. From every docking simulation, in which the ligand interacted with at least five known TCCD binding amino acids (ligand and amino acid distances were less than 4.5 Å), the complex with the ligand pose exhibiting the lowest binding energy was taken for further analysis. The TCCD binding amino acids at positions T289, H291, F295, P297, L308, L315, Y322, F324, I325, M340, F351, L353, A367, V381 and Q383 [55] (S1 Fig) were considered as the major residues defining the binding pocket (S2 Fig). 13 ligand molecules listed in Table 2 and shown in S3 Fig were selected for *in silico* docking based on their affinity to AhR ranging from high to zero affinity ligands. All other calculations and plotting not mentioned above were done in Python and R [56].

**Table 2:** Ligand molecules used in molecular docking.

Abbreviation	Name	PubChem ID	Equipotent concentration
TCDD [20]	Tetrachlorodibenzodioxin	15625	20 nM
TCDF [20]	2,3,7,8-tetrachlorodibenzofuran	39929	0.1 μM
Dibenzanthracene [20]	Dibenz-a,h-pyrene	5889	0.1 μM
Ficz [20]	5,11-dihydroindolo[3,2-b]carbazole-6-carbaldehyde	1863	0.1 μM
3mc [20]	Methylcholanthrene	1674	1 μM
Benz-a-pyrene [20]	Benz-a-pyrene	2336	1 μM
pbc126 [20]	3,4,5,3',4'-pentachlorobiphenyl	63090	1 μM
Bnf [20]	beta-Naphthoflavone	2361	1 μM
Indirubin [20]	Indigopurpurin	5359405	1 μM
Leflunomid [79]	leflunomide	3899	>30 μM
Yh [20]	Mivotilate	148185	10 μM
Sto [72]	7-Oxo-7H-benzimidazo[2,1-a]benz[de]isoquinoline-3-carboxylic Acid	16760660	-*
BBQ [72]	17-amino-6-chloro-3,10,17-triazahexacyclo[13.6.2.0^2^,^10^.0^4^,^9^.0^12^,^22^.0^19^,^23^]tricosa-1(21),2,4,6,8,12(22),13,15(23),19-nonaene-11,16,18-trione	sketched	-*

*Not interacting

## Results and Discussion

### Properties of the AhR homology models are not strongly dependent on template structures

Since no high resolution experimental structure of AhR has been determined, we employed homology modeling. Two structural models were generated based on PAS domains using human HIF-2α (PDBID: 1P97) and mouse CLOCK (PDBID: 4F3L) structures as templates, as the former one is employed in most of the studies in the last decade and the latter one exhibits higher sequence similarity to the human AhR PAS-B. We refer to the homology models in the manuscript as AhR_HIF_ and AhR_CLOCK_, indicating the template structures in subscripts. As the template structures contained no bound molecule, the homology models should be considered as apo conformations. The structures of the models and templates are similar to each other with pairwise root mean squared deviation (RMSD) 0.711 Å and 0.525 Å for HIF-2α/AhR_HIF_ and CLOCK/AhR_CLOCK_, respectively (S1 Fig). Our models were validated through protein structure evaluating methods including PROCHECK [41] and ProSA [39]. PROCHECK indicated amino acids in favored and disallowed Ramachandran regions as 76.5% and 3.9% for HIF-2α, 89.3% and 0% for CLOCK, 82.3% and 0% for AhR_HIF_, and 85.4% and 0% for AhR_CLOCK_, respectively. In contrast, the z-scores obtained using the ProSA web server were -6.53 for HIF-2α, -5.52 for CLOCK, -4.61 for AhR_HIF_, and -4.46 for AhR_CLOCK_, respectively, where a lower value indicates a better model. All these values are within the range for native protein structures of similar size. In addition we also estimated the quality of the models by the QMEAN server (Fig C in S1 Fig), in which the HIF-2α-based AhR model performed slightly better.

**Fig 2:**
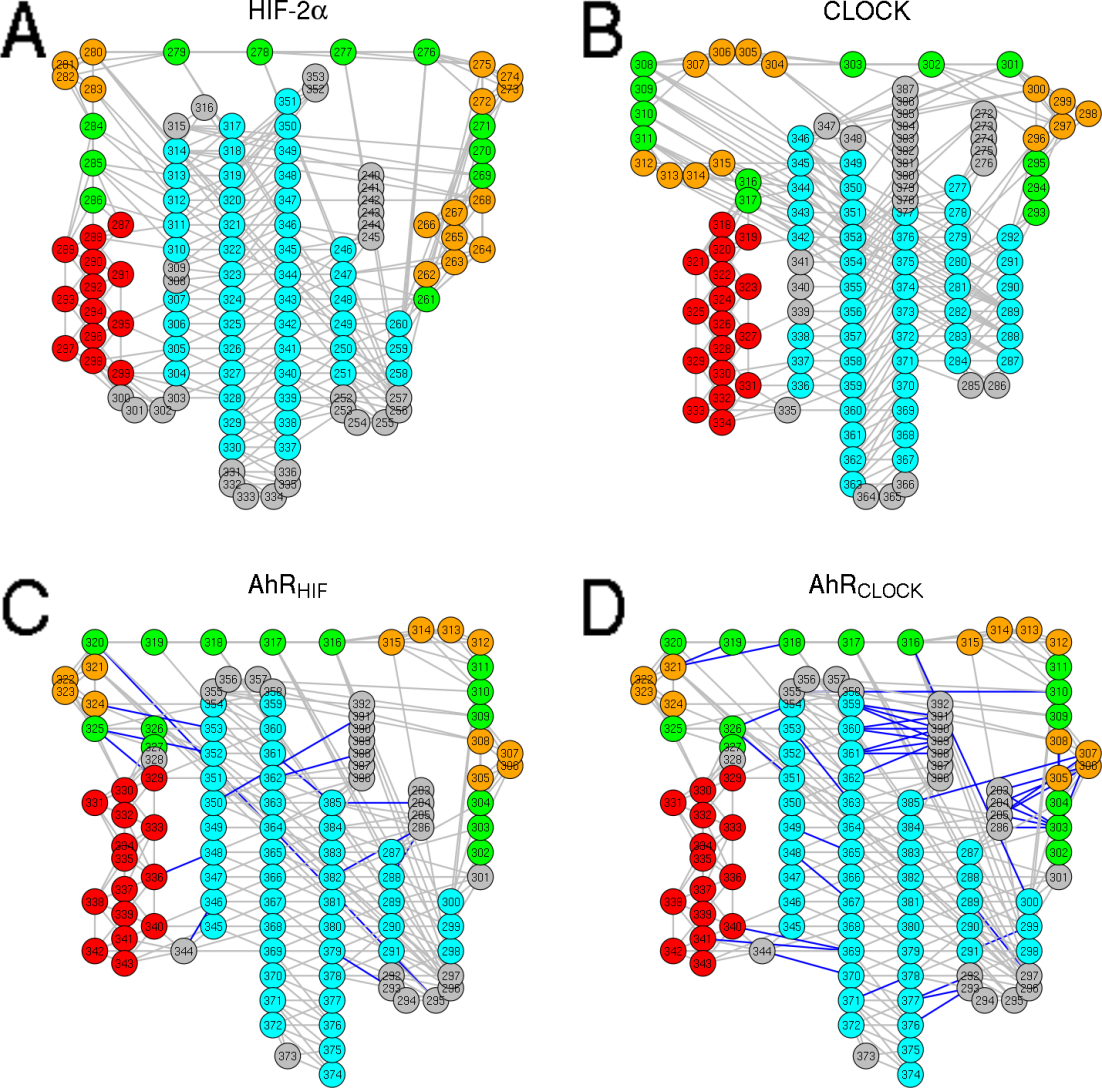
The contact maps of the homology models based on different templates are similar. The network of the interacting residues was determined by the Elastic Network Analysis Module of the MDAnalysis toolkit for the HIF-2α (A) and CLOCK (B) X-ray structures and AhR homology models based on the HIF-2α (C) and CLOCK (D) templates. Nodes are colored according to the main secondary structural modules in AhR (cyan: β-sheet, red: α-helix, green: ‘belt’ region, orange: small helices in the ‘belt’ region). On the C and D panels the blue edges represent interactions specific to either AhR_HIF_ or AhR_CLOCK_ compared to each other.

However, because of the low sequence similarity of PAS domains, we found it important to further characterize the homology models. To uncover potential structural differences between the homology models based on different templates which may influence the results of molecular dynamics simulations, we calculated the interaction graph of the template structures and homology models (Fig 2). The CLOCK structure exhibits a higher number of intramolecular interactions compared to the HIF-2α structure, especially the belt region (a region between the second β-sheet and the connector helix, Fig 1) provides more contacts to the β-sheet. Interestingly, the homology models possess highly similar interaction matrices which are more similar to that of CLOCK. Based on these observations, the stability of the two AhR models and the CLOCK structure can be predicted to be higher compared to the stability of the HIF-2α PAS-B structure.

**Fig 3:**
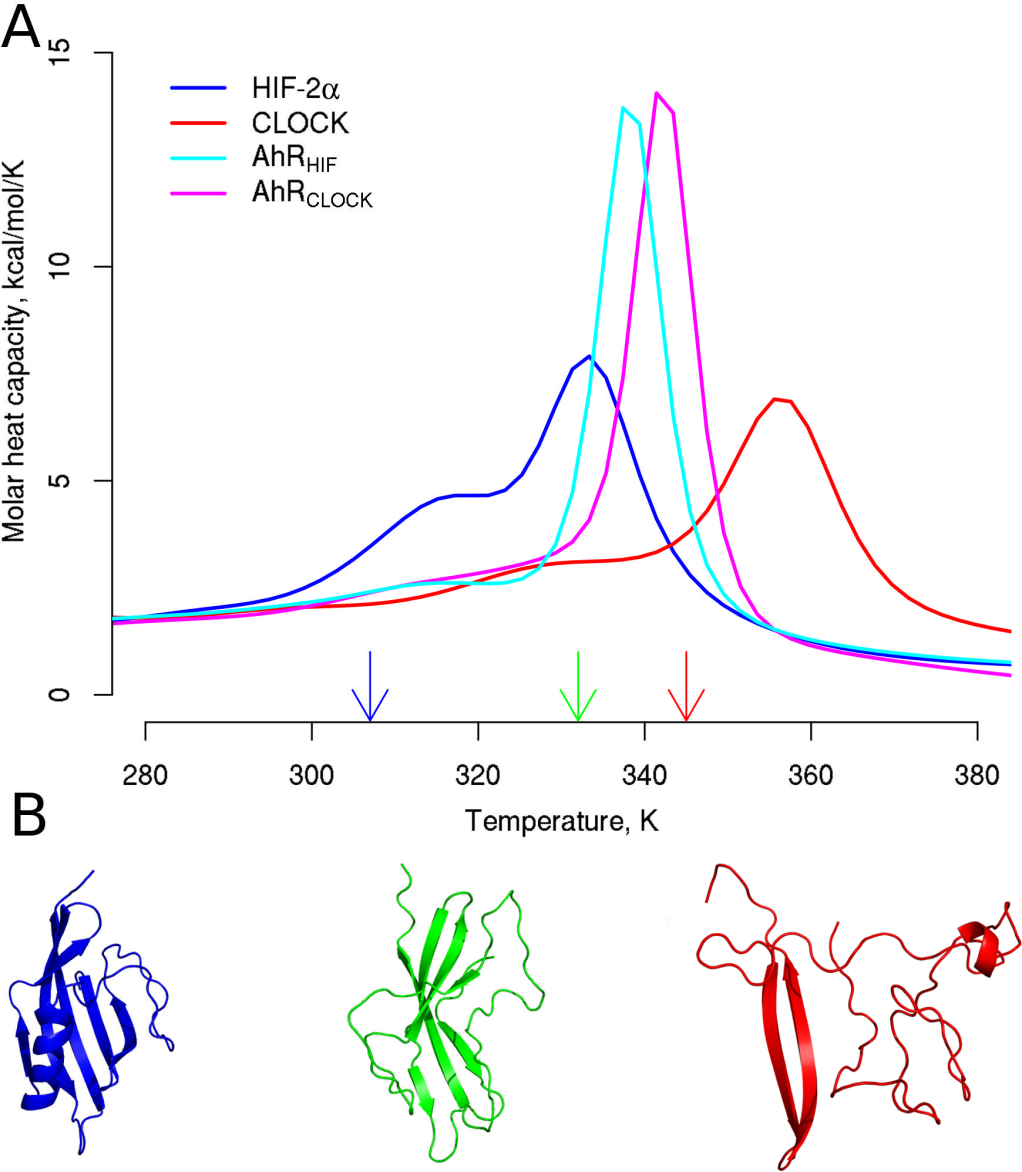
Melting temperatures of the homology models are between those of the templates. (A) Melting points as maxima of heat capacity curves were calculated from replica exchange DMD simulations using WHAM. (B) The unfolding events connected to the two peaks were determined and representative AhR_CLOCK_ conformations observed realized below and above the melting temperatures are shown as examples. The presented conformations are centroids of the largest clusters generated by clustering based on pairwise RMSD of conformations visited at 0.6116, 0.6604, and 0.6862 temperature units (indicated by arrows).

In order to test this and since the stability of the structures used in molecular dynamics simulations is also critical, we compared the melting temperature of the models, the templates, and also other PAS domains (Fig 3 and S4 Fig) employing replica exchange (RX) simulations using discrete molecular dynamics (DMD) [51,57,58]. In RX simulations several replicas run in parallel at different temperatures. Temperatures of replicas are exchanged in a stochastic manner based on the Metropolis criterion to ensure that replicas can escape from a local minimum at a higher temperature leading to an increased sampling of the conformational space. Although replica exchange itself provides a better sampling of the potential energy surface, we combined it with DMD to further improve computational performance.

Fig 3 displays the molar heat capacity versus temperature graphs, which are the first derivatives of the melting curves, providing information on the stability of the two AhR homology models and their templates (HIF-2α and CLOCK). Thus the peaks of the curves correspond to the melting temperatures, where folded and unfolded states of domains or subdomains are in equilibrium. The lowest melting point (0.6624 temperature units, 333K) can be observed for the HIF-2α structure. The two homology models exhibit quite similar melting points, 0.6704 temperature units (337K) for AhR_HIF_ and 0.6785 temperature units (341K) for AhR_CLOCK_, and the CLOCK structure is the most stable with a melting point of 0.7066 temperature units (356K). These values are in good accordance with the stability rank predicted by the contact maps (see above). Moreover, the melting curves indicate a two-step unfolding process by the more or less pronounced shoulders and a well-defined peak at higher temperatures which correspond to the unfolding of the connector helix and the β-sheets, respectively (Fig 3). The ‘belt’ region exhibit instability already at temperatures below the first observable melting point.

These results indicate that in spite of the low sequence similarity of PAS domains the main structural and thermodynamic properties of homology models based on different templates are similar to each other and also to those of other PAS domains with experimental high resolution structures (S4 Fig). Therefore and because the previously published AhR homology models were based on of HIF-2α, we employed both homology models (AhR_HIF_ and AhR_CLOCK_) throughout our study.

### Generation of conformation ensembles for *in silico* docking

Since docking to protein structures having flexible side chains or backbone is computationally highly intensive, we aimed to circumvent this problem by generating a large set of conformations and using them as targets in rigid docking calculations [59]. Using apo structures for such calculations is also justified, as PAS domains most likely recognize their ligands by conformational selection, as demonstrated for HIF-2α [40]. According to the theory of conformational selection, all possible conformations of the protein are realized in the absence of ligands including conformations being capable to bind substrates [60–63]. The substrate selects its favored conformation to bind to.

**Fig 4:**
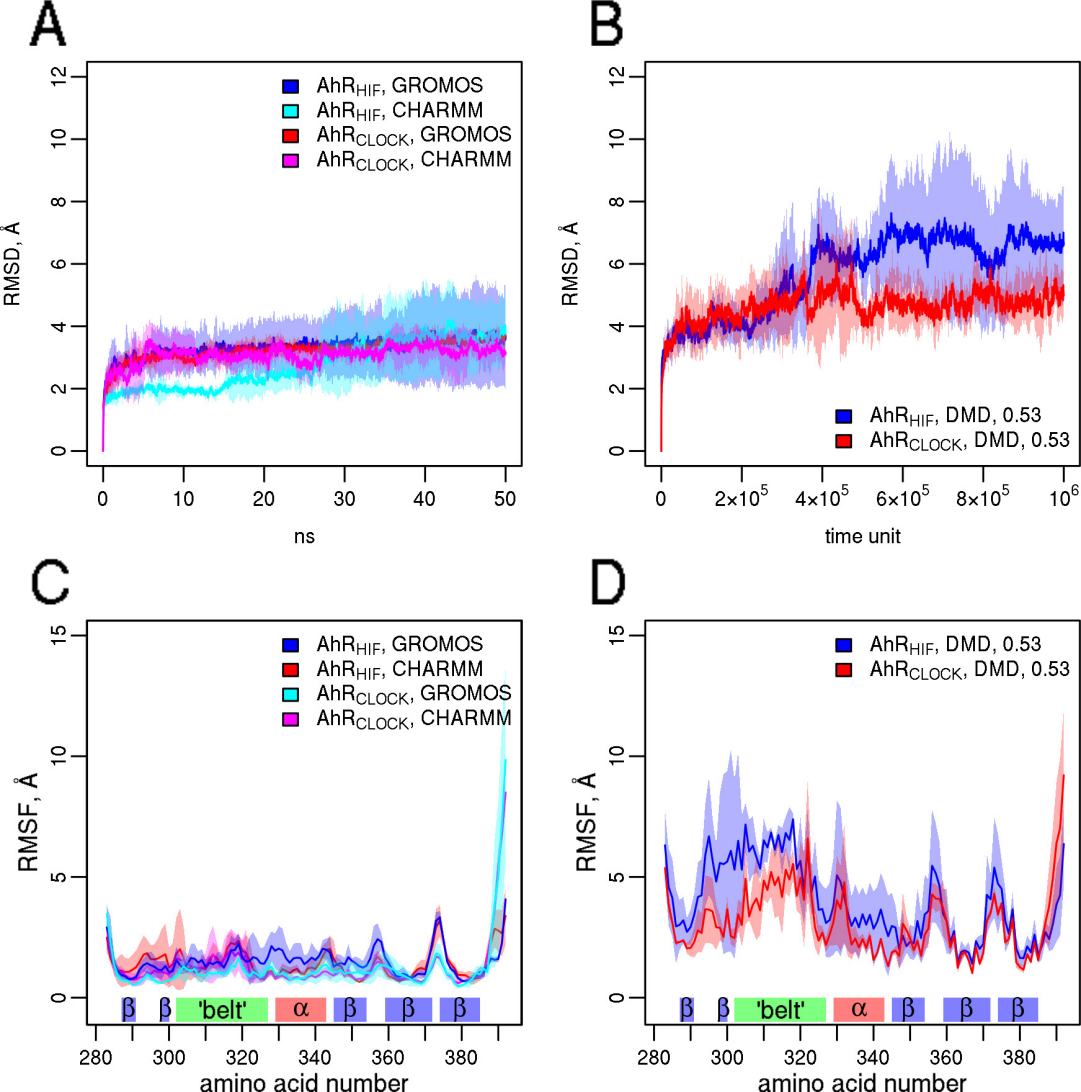
The belt region and the loops between secondary structural elements are the most flexible. Equilibrium simulations were performed using AhR_HIF_ and AhR_CLOCK_ models employing GROMOS and CHARMM force fields and discrete molecular dynamics (DMD, 0.53 temperature units). (A, B) Deviation from the starting conformation is characterized by RMSD values. Curves show the average value and colored bands correspond to the standard deviation from 3 independent simulations. (C, D) RMSF is calculated for individual residues for the characterization of protein flexibility.

Equilibrium simulations with the AhR homology models and their template structures were performed with three different simulation setups including GROMACS with two different force fields (GROMOS43a2 and CHARMM36) and πDMD. We employed the GROMOS force field for comparability, as it has been used in earlier studies of PAS domains [27]. We also utilized πDMD in spite of its simplified nature (discrete energy function and implicit water model are employed), since its algorithm and force field have been reported to achieve sampling quality and folding accuracy comparable to explicit-solvent simulations [49]. Its Medusa force field is based on CHARMM [49,50]. Importantly, DMD ensembles have been applied successfully in various docking studies [48,64–66]. Fig 4 shows the deviation of the homology models from the initial structure during the simulation (root mean squared deviation, RMSD) and also the fluctuations projected to individual residues (root mean squared fluctuation, RMSF). Fig 4A and 4B indicate that the two homology models exhibited similar level of deviation from the starting structure in conventional MD simulations, while the deviation from the initial conformations was higher in the case of DMD simulations and the dynamics of AhR_CLOCK_ was slightly decreased compared to AhR_HIF_ (Fig 4B). The RMSF values highlight the flexibility of the belt region as well as of the loops between the β-strands (Fig 4C and 4D) which are in good agreement with published MD simulation results using the HIF-2α PAS domain [32,66]. The higher mobility of the belt region may play a role in regulating the access to the binding pocket as also proposed by others [28,67].

A similar phenomenon has been suggested to be responsible for promiscuity of certain nuclear receptors and CYPs. Their ability to bind multiple ligands or substrates has been connected to their highly flexible thus polymorphic binding pocket [68]. To test whether this is the case for AhR, we performed equilibrium DMD simulations at two temperatures (0.53 and 0.59 temperature units) with PAS domains from proteins with or without ligand binding function, listed in Table 1. Comparing the average RMSF values of the ligand binding PAS domains to those PAS domains that are not known to participate in ligand binding, we could observe slightly but significantly higher average RMSF values for those structures that have ligand binding function (S5 Fig). Moreover, unfolding events have also been detected when transitioning between the holo and apo forms of HIF-2α [32]. Therefore we also compared the thermodynamic stability of a few PAS domains having experimentally determined structures and found that those domains exhibit somewhat higher stability which include a prosthetic group or do not have an explicit ligand binding function (S4 Fig).

These observations indicate that the ligand binding ability of PAS domains may require higher inherent flexibility. However, the small difference suggests that the main ground of promiscuous ligand binding may be encoded at a different level of structure or dynamics.

**Fig 5:**
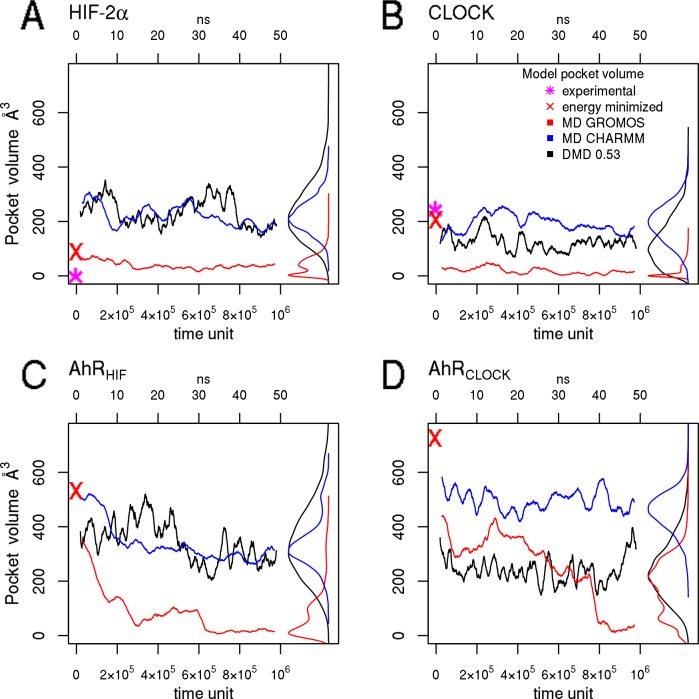
The size of the binding pocket is sufficiently large for ligand binding in CHARMM and DMD simulations, while significantly decreased or missing using the GROMOS force field. Average ligand binding pocket volume calculated for each conformation from 3–3 independent GROMOS, CHARMM, and DMD (0.53 temperature units) simulations are plotted for the HIF-2α (A), CLOCK (B), and AhR models (C, D). The distribution of the volume values are shown on the right of the graphs.

Therefore we continued to focus on and analyze the possible ligand binding pockets of the conformations generated by equilibrium simulations of the homology models and the corresponding templates. Fig 5 shows the volume of ligand binding pockets throughout MD and DMD simulations calculated by Fpocket2 [52]. The template structures exhibit relatively small internal cavities (90 Å^3^ for HIF-2α and 200 Å^3^ for CLOCK) compared to that of the homology models (400–600 Å^3^). The AhR PAS-B models exhibit larger voids because the amino acids surrounding their cavities possess smaller side chains (*e*.*g*. I325 in AhR is smaller than residues at homologous positions including Y281 in HIF-2α and Y313 in CLOCK). At a first glance strong force field dependence of the volume of the cavity can be observed. The GROMOS43a2 force field yielded small pockets mostly below 220 Å^3^ which is the approximate size of the TCDD molecule. In contrast, simulations with the CHARMM36 force field and DMD generated pockets with sizes sufficient for TCDD binding. Therefore conformations generated by MD employing CHARMM36 and DMD were used in docking simulations.

### Ligands with highly different affinity dock efficiently

In order to extract relevant information about the promiscuous binding properties of the AhR PAS-B domain, we carried out docking simulations. In the last years it has become evident that docking to a conformational ensemble compared to docking to a single structure resulted in more relevant ligand binding poses [3,14]. To some extent this approach overcomes the difficulties that are associated with taking the protein flexibility into account in docking simulations.

Docking of selected molecules was performed using Autodock Vina to every frame (2,500 from MD and 5,000 from DMD simulations). Moreover, docking simulations were carried out for all the three MD and three DMD simulation ensembles and the pose with the lowest energy from the relevant poses docked to each conformation was used in subsequent analysis. A pose was considered relevant if the ligand was within 4.5 Å to at least five of the 15 residues which take part in TCDD binding. These TCDD binding-fingerprint residues, which are listed in the Methods section, have been published to be responsible for ligand binding in the AhR PAS-B domain based on mutagenesis experiments [23,28,55]. Our approach for evaluation of ligand binding ensured that poses located outside of the pocket (e.g. on the outer side of the β-strands) were not taken into account. 13 molecules were selected for the docking simulations based on their affinity to AhR ranging from high affinity (equipotent concentration ≤ 1μM) to low affinity or non-binder ligands. Detailed information on the ligands is listed in Table 2 and their formulas are shown in S3 Fig.

**Fig 6:**
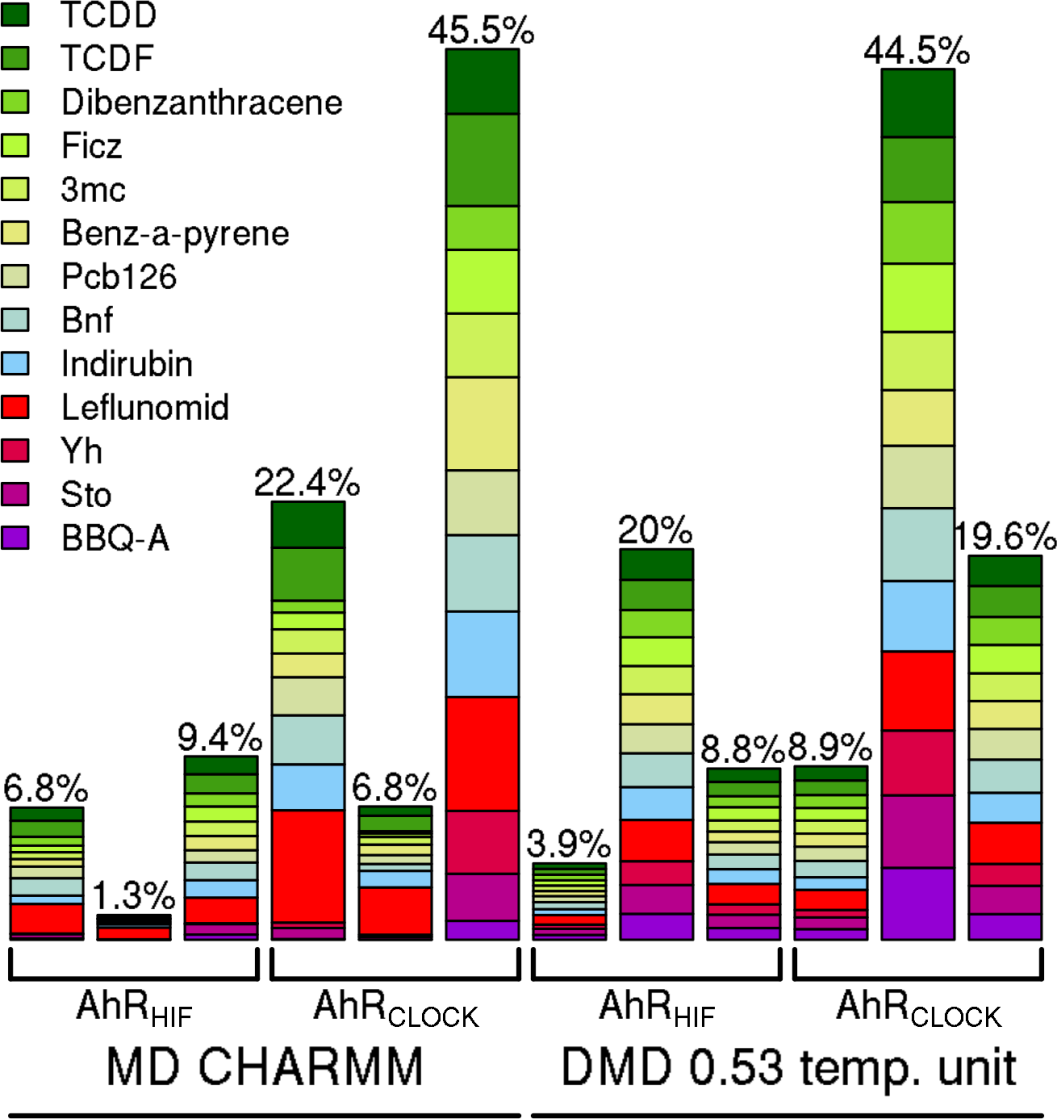
Docking to conformational ensembles does not show discriminative differences between binding of agonists with high and low affinity. Docking of molecules to each conformation from simulations was performed for three ensembles per setup using AutoDock Vina. The number of conformations that could accommodate a given molecule in its binding pocket was counted and depicted for each drug as a colored box with a size of that number. The percentage of these conformations compared to the possible maxima (2,500 and 5,000 in the case of MD and DMD, respectively) is indicated above the bars. Green colors indicate drugs with high affinity, while the other colors depict low affinity ligands or non-binders.

**Fig 7:**
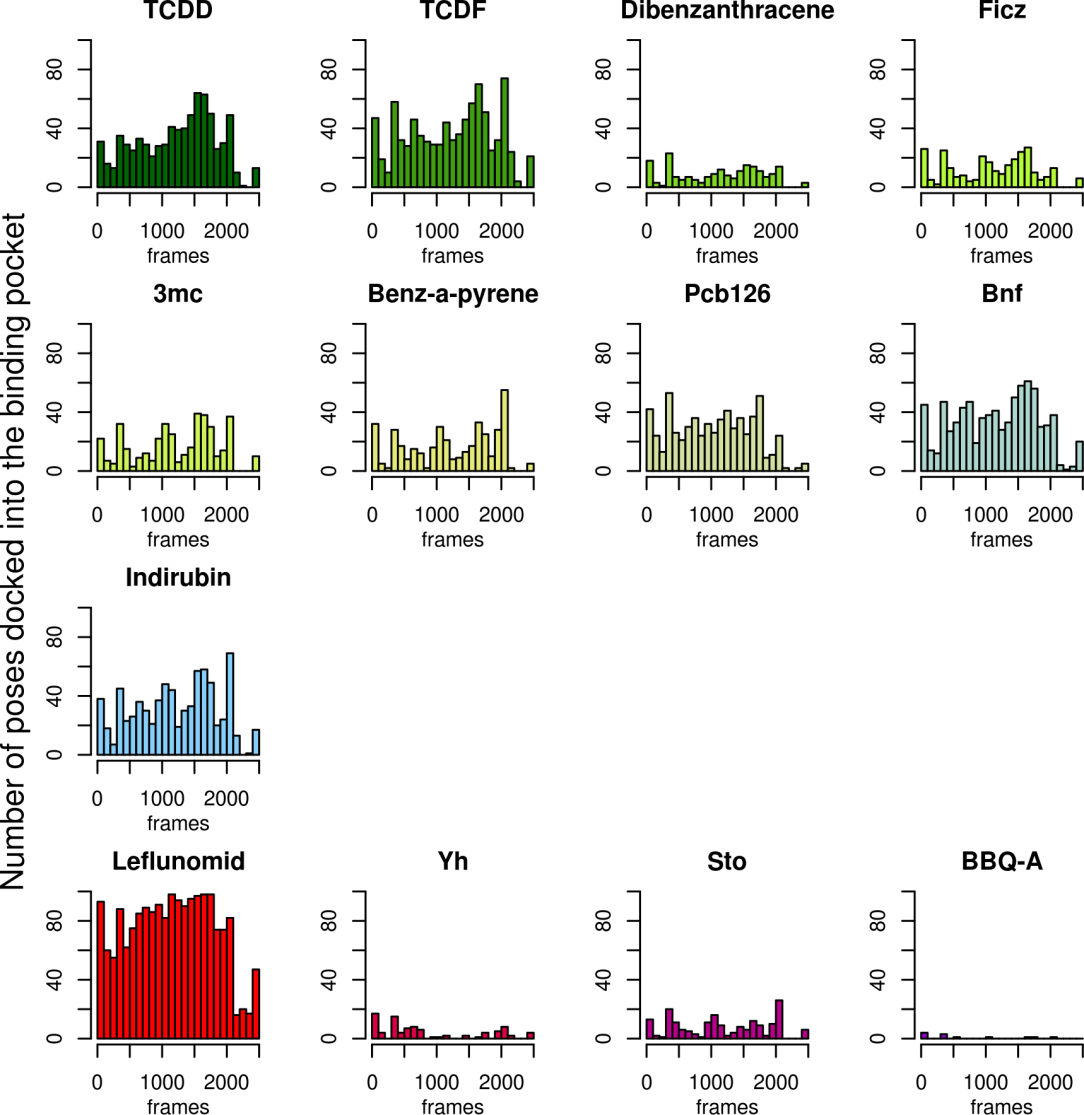
Low and high affinity ligands dock to similar set of frames. The number of conformations with relevant docking poses was counted in every ensemble, binned, and plotted for one of the MD CHARMM36 AhR_CLOCK_ simulations as an example. In this simulation the number of the relevant docked poses of low affinity molecules (except Leflunomid) is significantly lower. Green colors indicate drugs with high affinity, while the other colors depict low affinity ligands or non-binders. See Table 2 and S3 Fig for details on these molecules.

The relative docking success of AhR ligands with different affinities is plotted in Fig 6. The bar and the numbers above them indicate the percentage of the conformations that could accommodate a molecule inside the binding pocket. Unexpectedly, these total numbers of relevant poses are highly diverse in docking simulations on different ensembles, suggesting that MD simulations sampled different parts of the conformational space. In particular, most of the visited conformations by the HIF-2α-based AhR model were not sufficient to accommodate molecules in the binding pocket. Most importantly, molecules with high and low affinities could not be separated based on binding energies (S6 Fig). In addition, one of the lowest affinity molecules, Leflunomid binds the most efficiently, most likely because of its small size (212 Å^3^) that makes it easier to accommodate into the binding pocket. Interestingly, a smaller number of relevant binding poses could be observed for other low affinity ligands with AhR conformations derived from conventional MD simulations, except in the case of the AhR_CLOCK_ conformational ensemble with the highest number of relevant docking poses (Fig 6). In order to select conformations that accommodate high affinity but not low affinity ligands and vice versa, the number of relevant docking poses was projected to the ensembles to monitor conformations preferred by individual ligands. As an example, the AhR_CLOCK_ MD and DMD simulations are shown in Fig 7 and S7 Fig, respectively. The structure of the complexes revealed that three types of conformations exists in the ensembles: (1) one set binds only high affinity ligands in the pocket, (2) one set can accommodate all the tested molecules (both low and high affinity ligands), and (3) one group of conformations where ligands could only be docked on the surface of the PAS domain. Typical ligand binding poses and corresponding conformations are shown in S8 Fig. We analyzed the ensembles to highlight the characteristic properties of binding and non-binding conformations (S9 Fig) and to reveal side chains important for discriminating molecules with high and low affinities (S10 Fig). None of our efforts could uncover a characteristic set of residues with specific side chain orientations which would discriminate these groups of conformations thus low and high affinity ligands.

Since docking to conformational ensembles was not able to explain differences in binding of molecules with different affinities, we applied two more approaches. We also tested the novel Induced Fit Calculations software (Schrödinger 2015–3, [59,69]) that allowed flexibility not only for the ligand, but also for the protein. Although the extensive mode was applied, no differences in binding energies of molecules with high and low affinities could be observed (S11 Fig). Since bound molecules may have different effects on the PAS-B domain structure and dynamics as suggested for Bnf [20], we characterized the dynamics of drug-bound complexes using equilibrium DMD and MD simulations. However, we could not detect biologically relevant differences between complexes with high or low affinity ligands (S12 Fig).

All of the above results indicate that ligand binding pocket itself is not highly specific and may play only partial role in xenobiotics recognition. Prediction of ligand binding to the AhR PAS-B domain is challenging even when using conformational ensembles or cutting edge methods allowing protein flexibility.

### Ligand specificity may not be determined solely by PAS-B

Based on our results we speculated that ligand selection may be influenced by other proteins through protein-protein interactions happening in the cytoplasmic complex. Therefore we aimed to collect as much information on the AhR-containing complex as possible to assemble a structural model, which could be used to predict the mechanism of AhR ligand recognition. Binding location of cytoplasmic AhR binding partners were collected from the literature and projected on the AhR sequence (Fig 1). Hsp90 binds to both the ligand binding domain and also the N-terminal part of AhR involving the middle region of Hsp90 (amino acids 272–617) [10,70]. Although the available information is too sparse to generate a reliable Hsp90/AhR/XAP2 complex, it allowed generating a model for allosteric communications upon ligand binding. Since the binding initiates the translocation of the complex to the nucleus [10], this event also has to trigger the visibility of the nuclear localization signal (NLS), which can be found on the AhR N-terminus [18]. The signal from the PAS-B to the NLS is not expected to propagate internally through the AhR protein, as the linker regions between the domains are highly flexible. However, both the ligand binding PAS-B and the NLS are connected to the middle section of Hsp90, thus the information of ligand binding is very likely communicated allosterically to the NLS via Hsp90 (Fig 1). If a somewhat rigid structure is supposed for this communication, the role of p23 in the complex can be understood: this chaperone stabilizes Hsp90 in a rigid, ATP-bound conformation [10,15,71]. Another member of the cytoplasmic complex, XAP2 might also participate in the transduction of conformational changes associated with ligand binding, since it is bound to the C-terminus of PAS-B, where the central β-strand is located (Fig 1). This location is the most rigid part of PAS-B, thus even minor changes may dramatically influence the whole domain. Moreover, this part of PAS-B may serve as a ligand sensor, as it contains the residue Q383 penetrating into the middle of the ligand binding pocket.

## Conclusions

**Fig 8:**
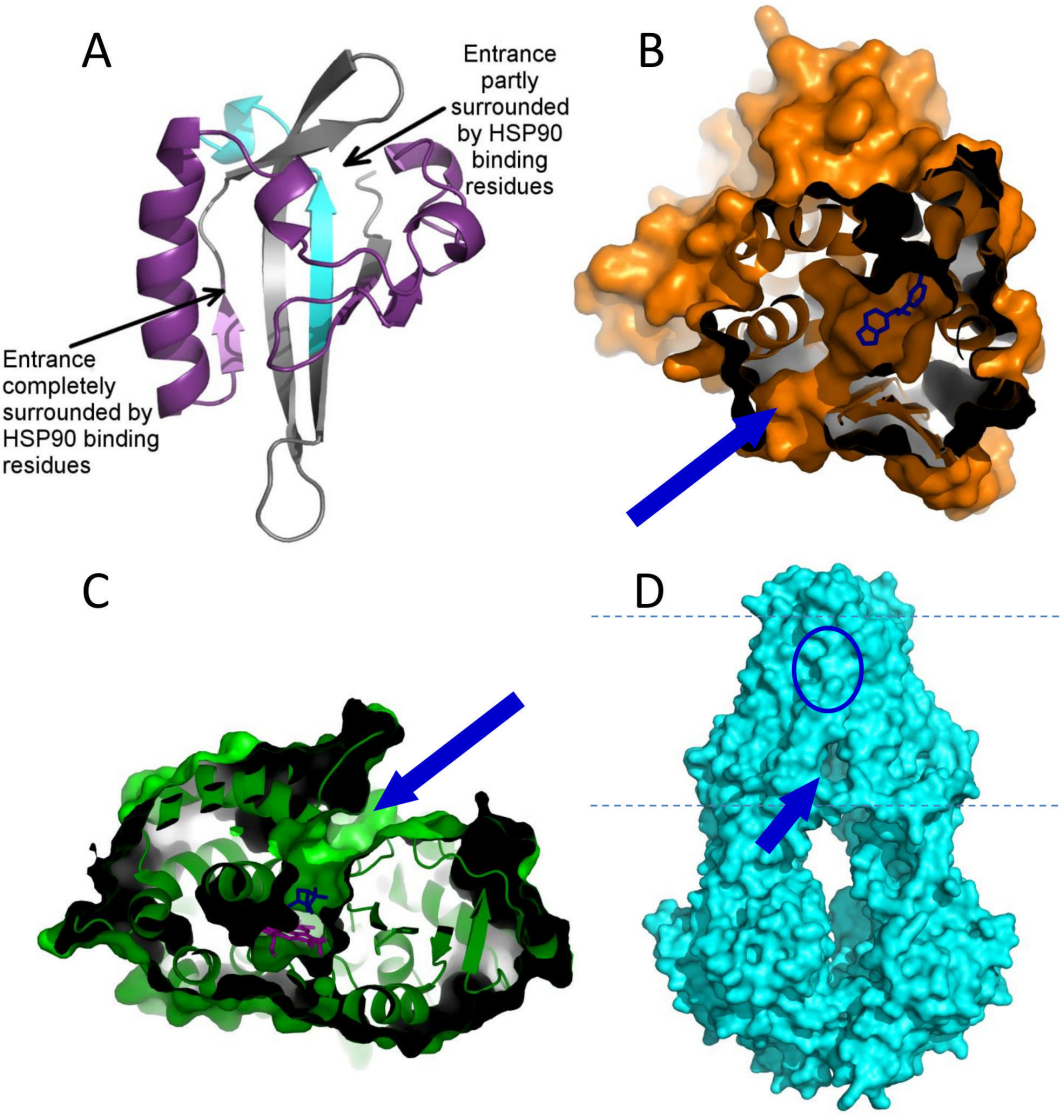
Discrimination of xenobiotics from endogenous molecules may be realized by access pathways to the binding pockets, acting as filters. (A) Based on visual examination of our simulations, two putative ligand entry pores (indicated with arrows) can be identified in the conformations of the AhR PAS B domain. Both of these regions are involved in Hsp90 binding (purple segments) suggesting that Hsp90 can directly influence the accessibility of the pores and potentially participate in ligand selection (cyan segment indicates XAP2 binding residues.) (B, C) A role of access pathways (indicated on the protein structures by blue arrows) in ligand selection has been described for nuclear receptors (B: PXR, PDBID:3R8D) and also for CYPs (C: bacterial CYP101D2, PDBID:3NV6). (D) Similarly, a substrate selective access pathway from the cytoplasmic leaflet of the membrane to the low affinity binding pocket of ABC multidrug transporters localized in the outer membrane leaflet may play a role in xenobiotics recognition (*T*. *maritima* TM287/288, PDBID:4Q4J). Blue arrows: ligand/substrate entry pathway; blue circle: location of the ligand binding pocket inside the structure.

We aimed to understand the molecular basis of multidrug recognition of large membrane transporters based on ligand interaction of the small soluble promiscuous PAS-B domain of AhR. Although amino acids surrounding the binding pocket have been shown by computational studies and mutagenesis [23,28,55] to play an important role in ligand selection, we could observe a relatively uniform docking of chemicals with highly different affinities employing extensive structural modeling and molecular dynamics simulations. The side chains protruding into the binding pocket certainly have effect on xenobiotics binding and can explain species differences in ligand recognition. However, this domain may play a more important role in allosteric signal transduction than in ligand selection, especially when taking into account the binding of various molecules into the same pocket as agonists or antagonists [20,72]. We hypothesize that the main step of ligand selection may happen by access channels connecting the domain surface to the binding pocket. A potential entrance is partially covered by the belt region, which is an interacting site of Hsp90, thus this chaperone can affect ligand accessibility [28,67] (Fig 8). Moreover, a different entrance path on the PAS-B can also be observed, which is indicated to be surrounded and directly controlled by Hsp90. The simulations of the complexes of drugs and PAS-B (S12 Fig) indicate that the segments with decreased dynamics overlap with both Hsp90 binding regions and the central β-sheet core of the structure (Fig 1). This result also strengthens the hypothesis that the ligand binding event and the visibility of the nuclear localization signal is connected by Hsp90, stabilized in the rigid conformation by p23.

A similar ligand selection mechanism via access channels have been proposed for other multidrug binding proteins, including CYPs and nuclear receptors [29–31] (Fig 8). However, in the case of AhR, the selection pathway may be formed not internally, but created by intermolecular interactions provided by the protein interaction partners. In a somewhat similar way, cofactor binding of nuclear receptors has been reported to influence their ligand access and ligand binding [31,44]. In the case of large multidrug transporters, selection of xenobiotics from endogenous molecules may also occur via filtering before entering the binding pocket (Fig 8). A specific orientation of transmembrane helices and their intracellular parts, which could be realized in a bottom-closed apo and not in a bottom-open apo transporter conformation [46,47], may serve as the structural basis of a substrate selection to differentiate harmful chemicals from non-toxic molecules.

In summary, promiscuous proteins may exhibit a hydrophobic binding pocket with low selectivity, and the differentiation of endogenous molecules from xenobiotics may be realized via an access channel from the outside of the molecule or protein complex to the internal binding cavity.

## Supporting Information

S1 Fig Homology modeling of AhR PAS-B. (PDF)

S2 Fig The ligand binding pocket of AhR PAS-B defined by residues interacting with TCCD. (PDF)

S3 Fig Compounds selected for in silico docking. See Table 2 for more details.
(PDF)

S4 Fig Bound molecules increase PAS domain stability. (PDF)

S5 Fig Ligand binding PAS domains exhibit higher fluctuations compared to PAS domains without a ligand binding function. (PDF)

S6 Fig In silico binding energies do not correlate with experimental ligand affinities. (PDF)

S7 Fig Conformations generated by DMD cannot effectively differentiate ligands with different affinity similarly to ensembles from conventional MD simulations. (PDF)

S8 Fig The AhR PAS-B domain can reside in conformations that either (1) bind only high affinity ligands, (2) are capable of binding all the tested molecules, or (3) unable to bind any molecules in the binding pocket. (PDF)

S9 Fig Protrusion of the amino acids delineating the pocket modulates ligand binding. (PDF)

S10 Fig TYR322 and HIS337 may play a role in differentiating molecules with high and low affinities. (PDF)

S11 Fig Induced Fit calculations cannot differentiate between molecules with high and low affinities. (PDF)

S12 Fig Complexes with high and low affinity molecules behave similarly. (PDF)
