# Supplementary material for: Access Path to the Ligand Binding Pocket May Play a Role in Xenobiotics Selection by AhR
Source: PLoS One. 2016 Jan 4;11(1):e0146066. doi: 10.1371/journal.pone.0146066 (PMC4699818; doi:10.1371/journal.pone.0146066)
Supplement: S1 Fig — (PDF) [file pone.0146066.s001.pdf]

S1 Fig. Homology modeling of AhR PAS-B. A: Sequence alignment of HIF-2α, CLOCK, human AhR and mouse AhR PAS-B sequences was generated by ClustalW. Sequence numbering corresponds to the human AhR sequence. Green circles indicate amino acids taking part in TCDD binding, which was determined in mutational experiments for the mouse AhR by Pandini *et al.* [1]. B: The best homology models display an RMSD of 0.711 Å and 0.525 Å after structural alignment to their templates for AhR_{HIF} and AhR_{CLOCK}, respectively (HIF-2α, CLOCK, AhR_{HIF} and AhR_{CLOCK}). C: Model quality was also estimated by the QMEAN server (http://swissmodel.expasy.org/qmean/cgi/index.cgi; [2]) beside ProSA [3] and ProCheck [4]. The estimated quality by QMEAN is slightly lower for the generated structures compared to that of the experimental structures.

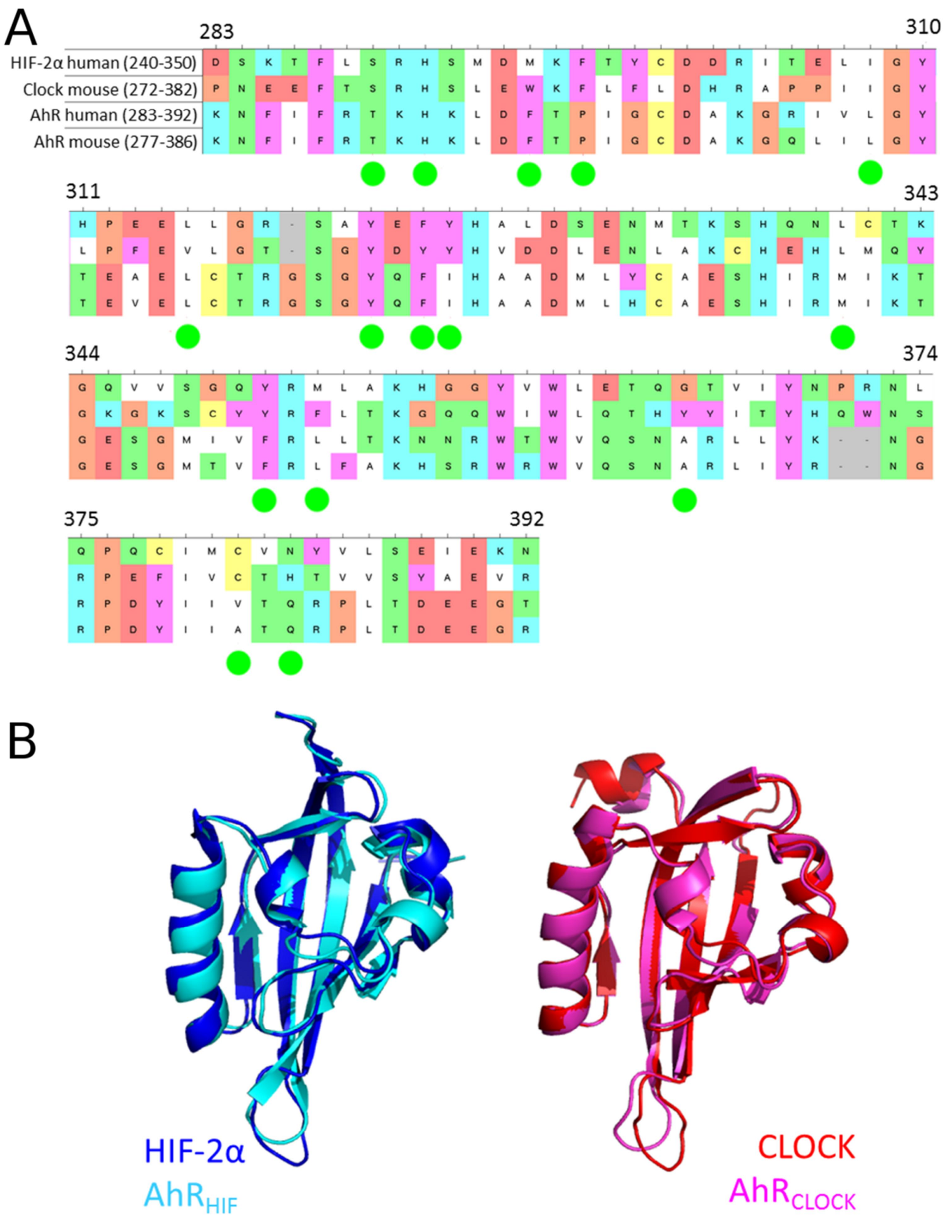

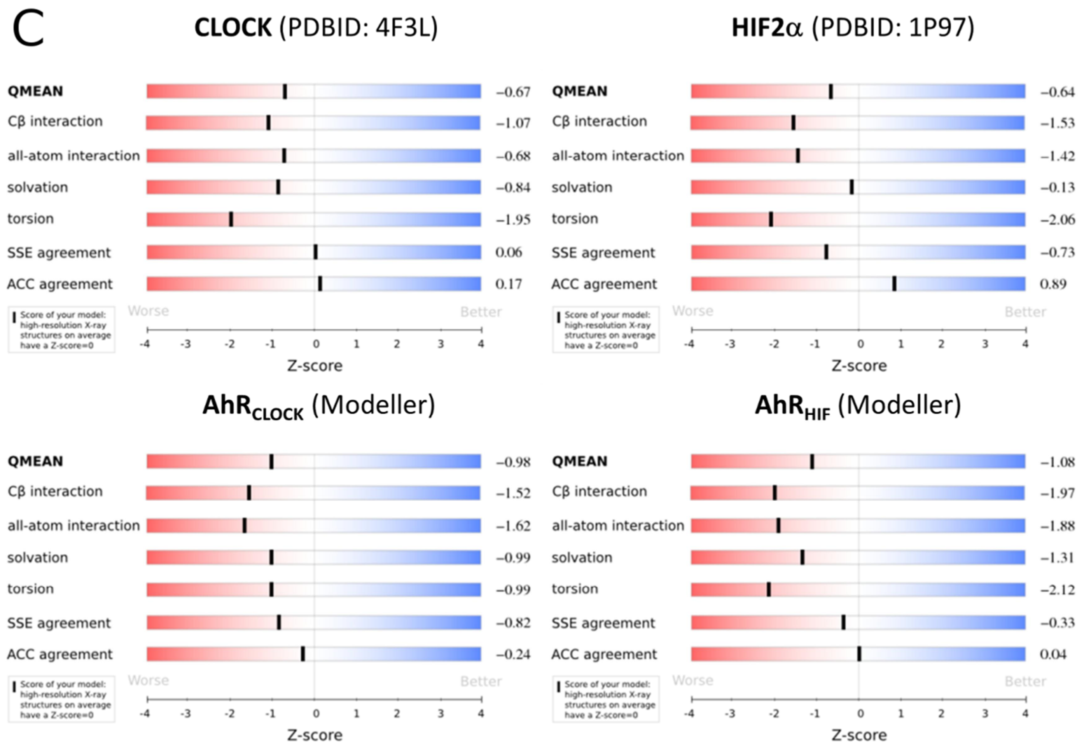

C
CLOCK (PDBID: 4F3L)
HIF2α (PDBID: 1P97)
AhR CLOCK (Modeller)
AhR HIF (Modeller)
QMEAN
Cβ interaction
all-atom interaction
solvation
torsion
SSE agreement
ACC agreement
Worse
Better
Z-score
Score of your model: high-resolution X-ray structures on average have a Z-score=0
−0.67
−1.07
−0.68
−0.84
−1.95
0.06
0.17
−0.64
−1.53
−1.42
−0.13
−2.06
−0.73
0.89
−0.98
−1.52
−1.62
−0.99
−0.99
−0.82
−0.24
−1.08
−1.97
−1.88
−1.31
−2.12
−0.33
0.04
