# Supplementary material for: Access Path to the Ligand Binding Pocket May Play a Role in Xenobiotics Selection by AhR
Source: PLoS One. 2016 Jan 4;11(1):e0146066. doi: 10.1371/journal.pone.0146066 (PMC4699818; doi:10.1371/journal.pone.0146066)

S2 Fig. The ligand binding pocket of AhR PAS-B defined by residues interacting with TCCD. Blue stick representation: residues interacting with TCCD based on a mouse AhR homology model [1]; gray box: the search space (whole domain) defined for *in silico* docking.

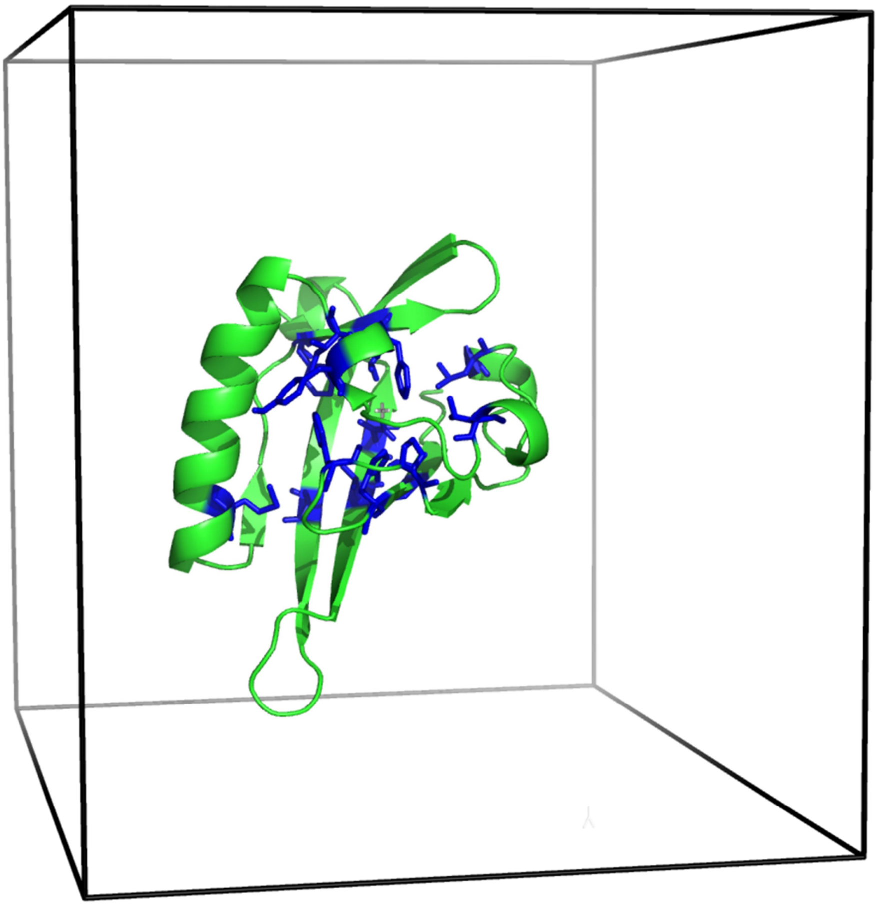

Supplement: S2 Fig — (PDF) [file pone.0146066.s002.pdf]
