# Supplementary figures and images for: Access Path to the Ligand Binding Pocket May Play a Role in Xenobiotics Selection by AhR
Source: PLoS One. 2016 Jan 4;11(1):e0146066. doi: 10.1371/journal.pone.0146066 (PMC4699818; doi:10.1371/journal.pone.0146066)

**S3 Fig. Compounds selected for *in silico* docking.** See Table 2 for more details.

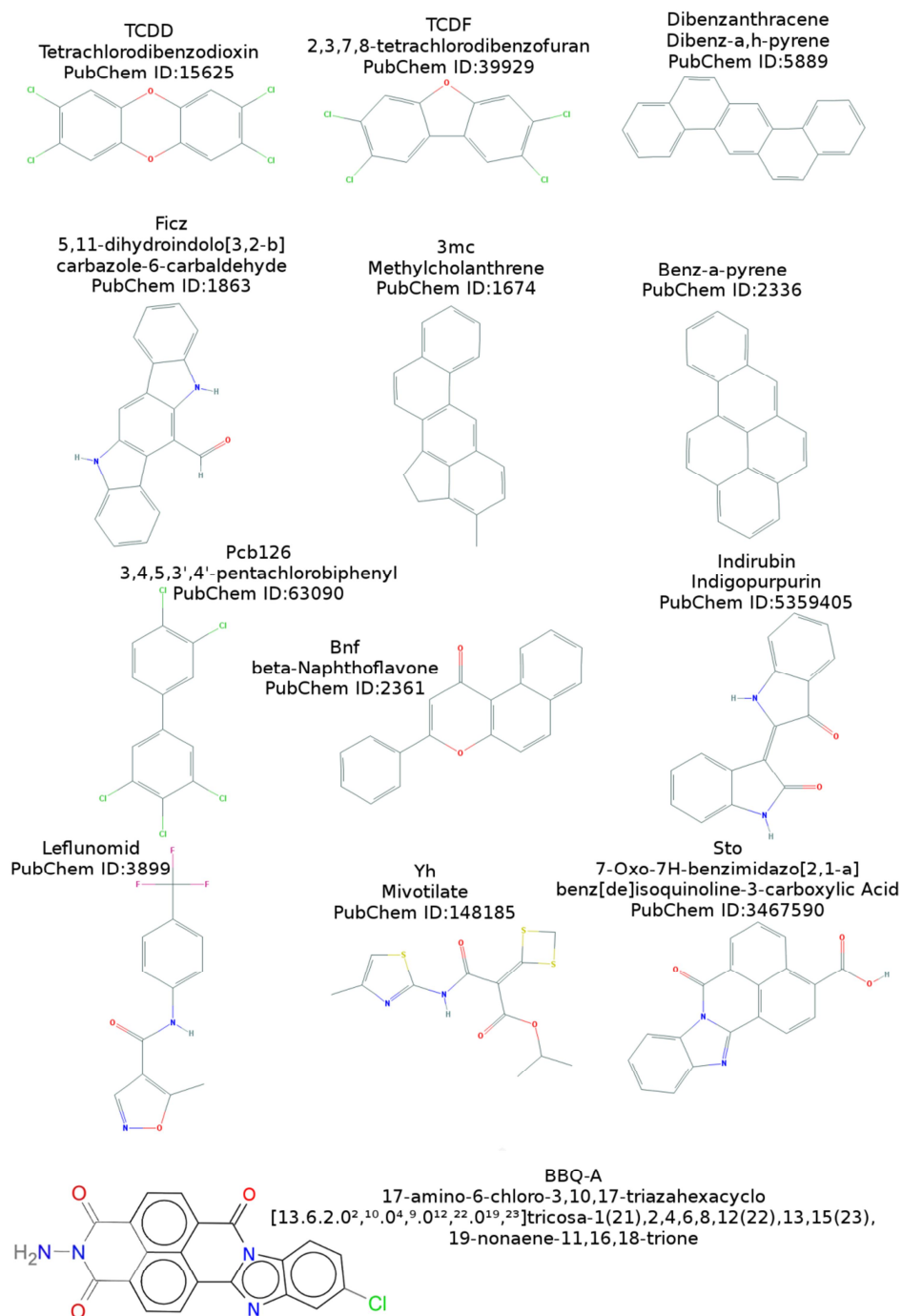

Supplement: S3 Fig — See Table 2 for more details. (PDF) [file pone.0146066.s003.pdf]
