# Supplementary material for: Access Path to the Ligand Binding Pocket May Play a Role in Xenobiotics Selection by AhR
Source: PLoS One. 2016 Jan 4;11(1):e0146066. doi: 10.1371/journal.pone.0146066 (PMC4699818; doi:10.1371/journal.pone.0146066)

S4 Fig. Bound molecules increase PAS domain stability. Melting points as maxima of heat capacity curves were calculated from replica exchange DMD simulations using WHAM for several other PAS domains. Melting temperatures from hERG to FixL are 0.7066 (356K), 0.6945 (349K), 0.6744 (339K), 0.6744 (339K) and 0.6704 (337K) respectively. The hERG PAS domain, which is not known to bind any ligand, has the highest melting point. The PYP PAS domain was simulated both in the presence and absence of its prostetic group. Apo PYP, apo HIF-2α, and apo FixL have the lowest melting points. We could not simulate holo FixL with its prostetic group hem, as Fe^{2+} ions are not incorporated into the force field of DMD. HIF-2α structures derived from X-ray (PDBID:3F1P, this graph) or NMR experiments (PDBID:1P97, Fig. 3) exhibit very similar melting temperatures. An increased stability is provided either by the intrinsic properties of the domain (e.g. in the case of hERG and CLOCK with no ligand binding properties) or by bound molecules (e.g. ligands or prostetic groups).

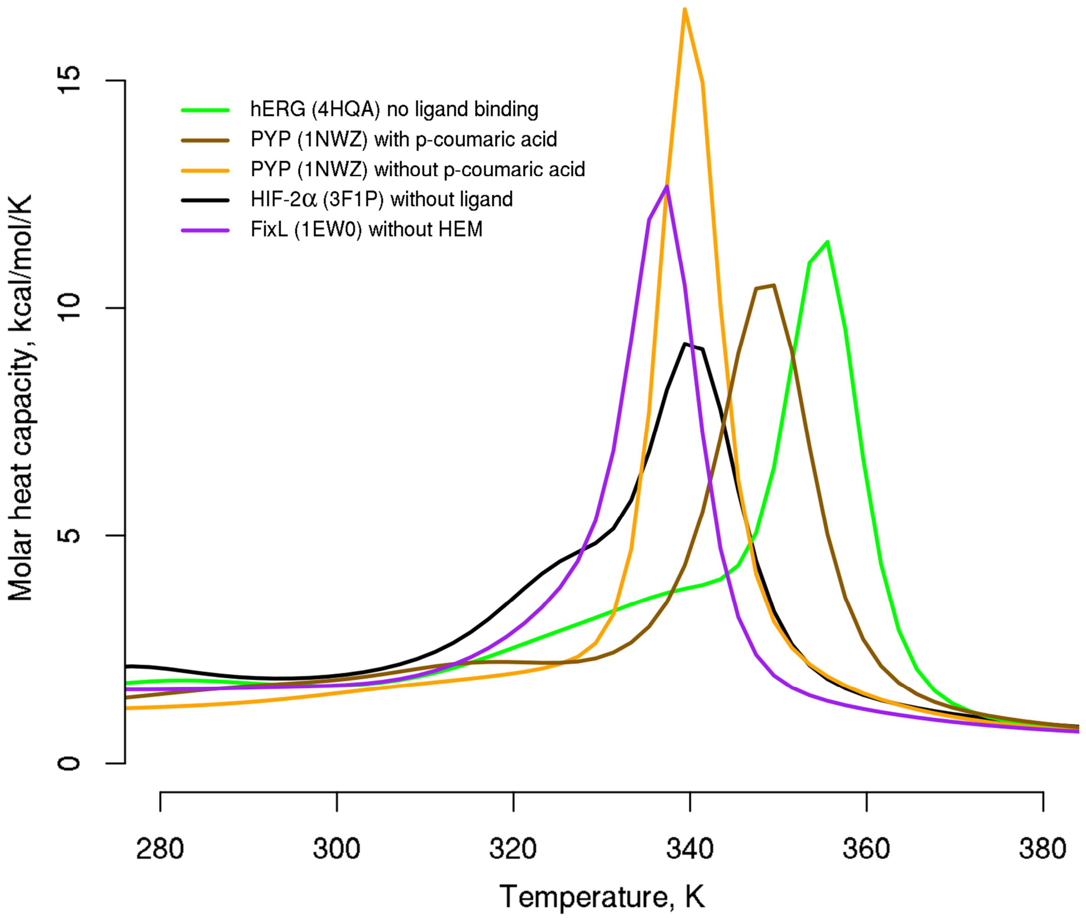

Supplement: S4 Fig — (PDF) [file pone.0146066.s004.pdf]
