# Supplementary material for: Access Path to the Ligand Binding Pocket May Play a Role in Xenobiotics Selection by AhR
Source: PLoS One. 2016 Jan 4;11(1):e0146066. doi: 10.1371/journal.pone.0146066 (PMC4699818; doi:10.1371/journal.pone.0146066)

S5 Fig. Ligand binding PAS domains exhibit higher fluctuations compared to PAS domains without a ligand binding function. DMD simulations with various PAS domains (see Table 1 for details) were performed for 1,000,000 time units at 0.53 and 0.59 temperature units. RMSF values were calculated along the trajectories (n=3 for each PAS domain) and projected on the structures. Warmer color and thicker representation indicate higher RMSF values. The RMSF of ligand binding PAS domains were slightly higher (t-test, difference=1.29 and 2.68 Å, p= 0.0521 and 0.0345 for simulations at 0.53 and 0.59 temperature units, respectively). Results from simulations at 0.59 temperature units are shown. Ligand binding structures are labeled by asterisk, the templates by number signs, and PAS domains with a prosthetic group by currency signs.

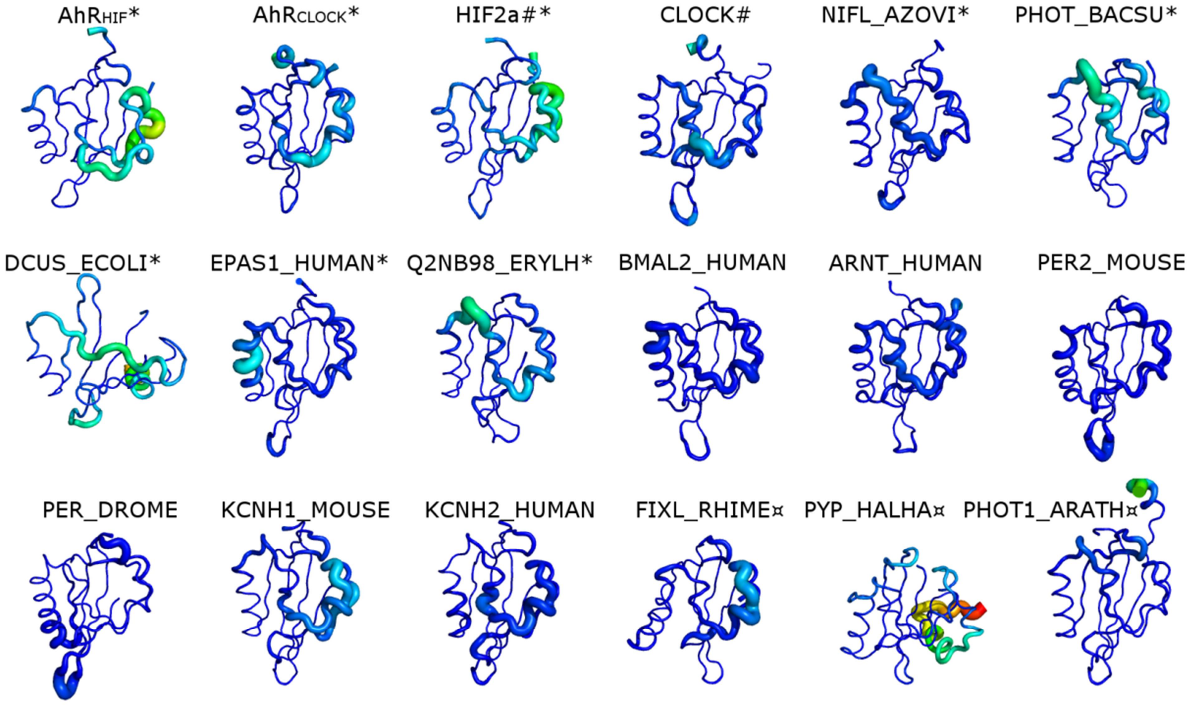

Supplement: S5 Fig — (PDF) [file pone.0146066.s005.pdf]
