# Supplementary material for: Access Path to the Ligand Binding Pocket May Play a Role in Xenobiotics Selection by AhR
Source: PLoS One. 2016 Jan 4;11(1):e0146066. doi: 10.1371/journal.pone.0146066 (PMC4699818; doi:10.1371/journal.pone.0146066)

S6 Fig. *In silico* binding energies do not correlate with experimental ligand affinities. Binding energies were extracted from the docking results of one of the full ensemble of AhR_{CLOCK} CHARMM36 simulations as an example. Density histograms of binding energies of ligands docked into the binding pocket (blue) and to the protein surface (red) were compared. Binding energy values of ligands docked inside are consistently lower than that of docked outside. Importantly, these *in silico* affinities do not correlate with experimental ones, e.g. many low affinity ligands exhibit higher *in silico* affinity than that of TCDD.

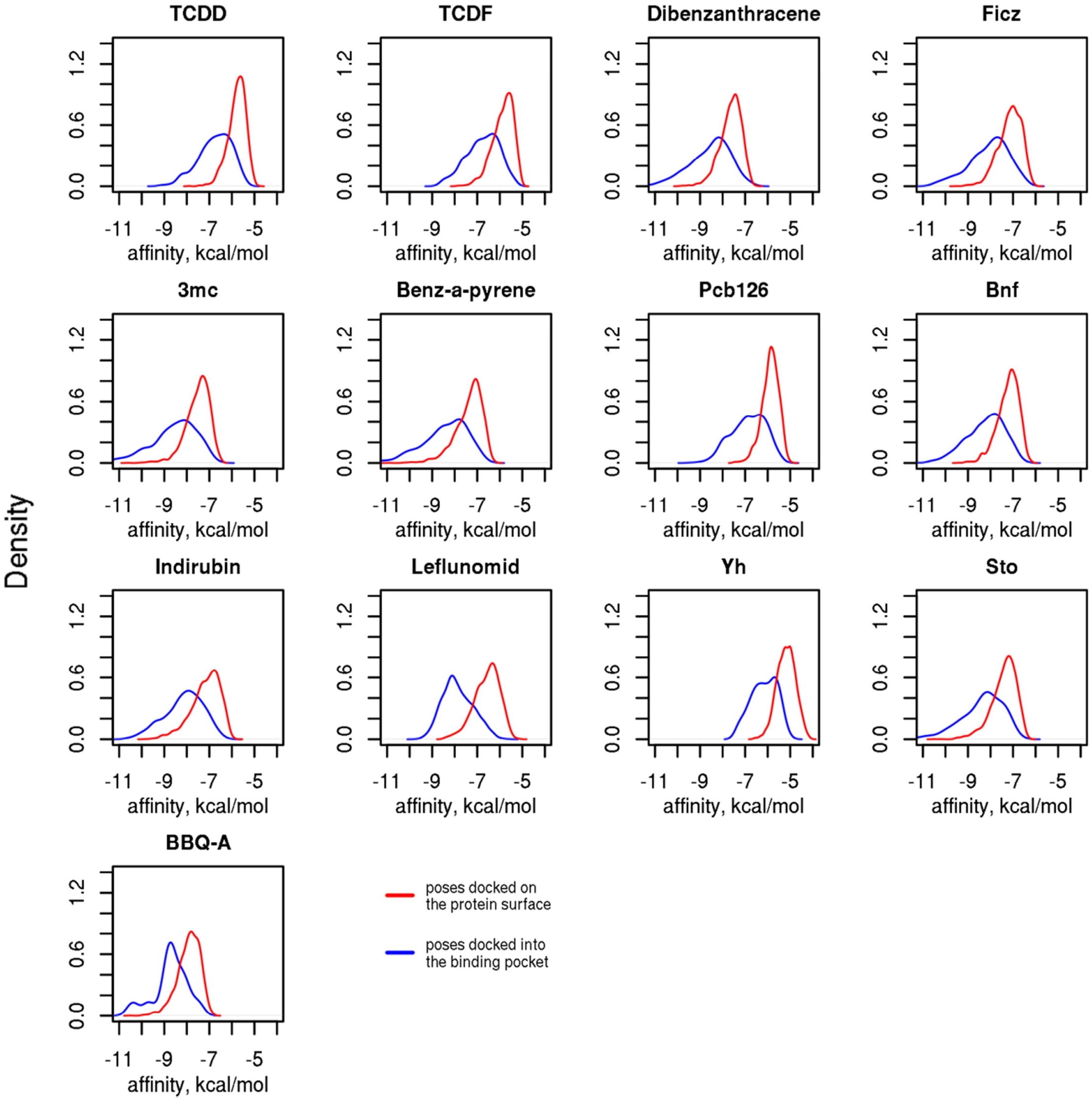

Supplement: S6 Fig — (PDF) [file pone.0146066.s006.pdf]
