# Supplementary material for: Access Path to the Ligand Binding Pocket May Play a Role in Xenobiotics Selection by AhR
Source: PLoS One. 2016 Jan 4;11(1):e0146066. doi: 10.1371/journal.pone.0146066 (PMC4699818; doi:10.1371/journal.pone.0146066)

S7 Fig. Conformations generated by DMD cannot effectively differentiate ligands with different affinity similarly to ensembles from conventional MD simulations. The number of conformations with relevant docking poses was counted in every ensemble, binned, and plotted. Similar to calculations from MD simulations (Fig. 7), the numbers of relevant binding events are slightly smaller for molecules with low affinity. Here, the AhR_{CLOCK} ensemble generated by DMD at 0.53 temperature units is shown as an example. Similar results were obtained for the DMD ensemble using AhR_{HIF} and also for ensembles generated using either model in simulations at 0.59 temperature units (not shown). Green colors indicate drugs with high affinity, while the other colors depict low affinity ligands or non-binders.

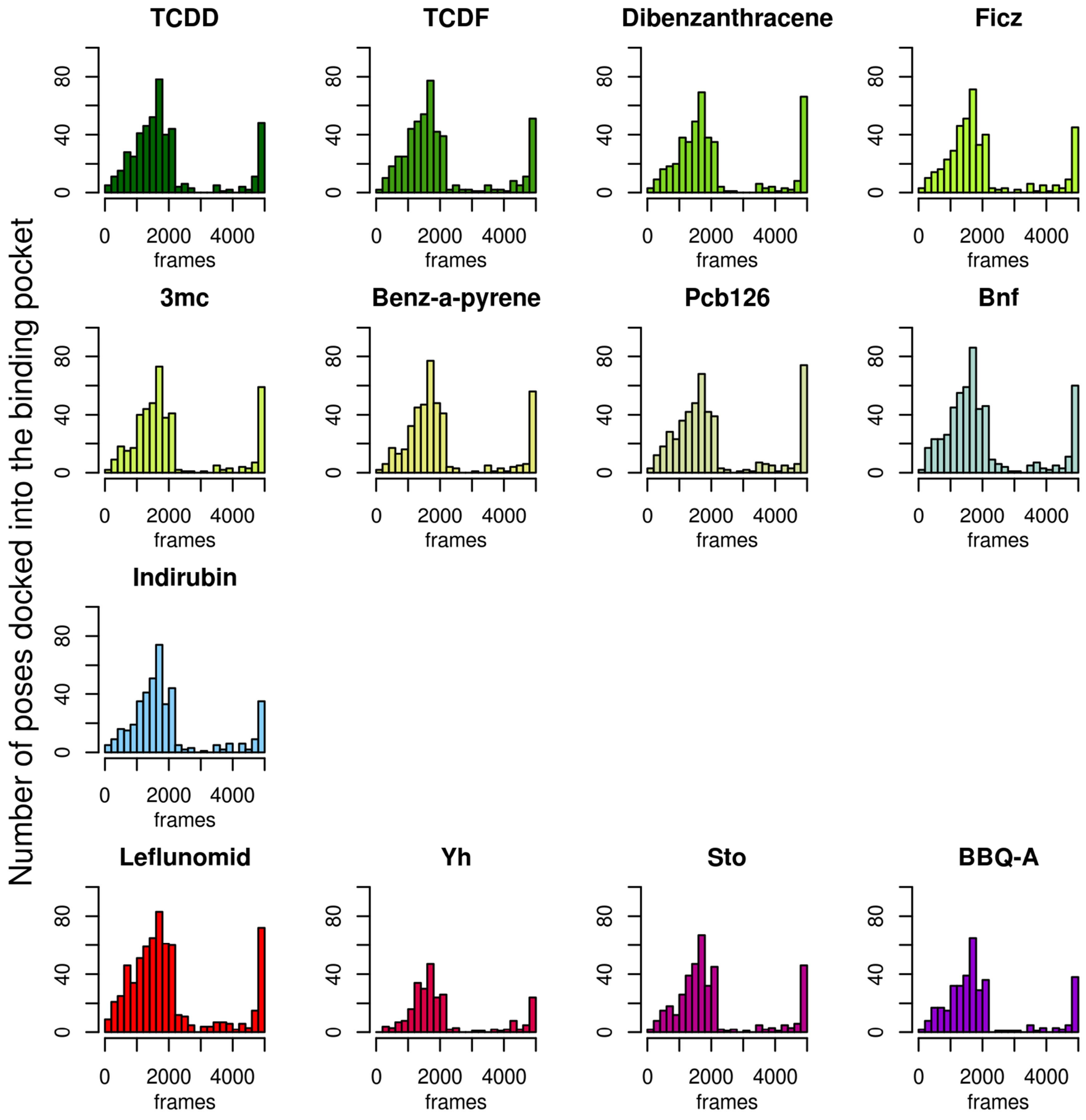

Supplement: S7 Fig — (PDF) [file pone.0146066.s007.pdf]
