# Supplementary material for: Access Path to the Ligand Binding Pocket May Play a Role in Xenobiotics Selection by AhR
Source: PLoS One. 2016 Jan 4;11(1):e0146066. doi: 10.1371/journal.pone.0146066 (PMC4699818; doi:10.1371/journal.pone.0146066)

S8 Fig. The AhR PAS-B domain can reside in conformations that either (1) bind only high affinity ligands, (2) are capable of binding all the tested molecules, or (3) unable to bind any molecules in the binding pocket. Two AhR_{CLOCK} conformations were taken as examples from simulations with CHARMM36. A: This selected conformation binds TCDD with the highest affinity (-9.2 kcal/mol; top). All molecules with high affinity bind into the pocket, while the low affinity ligands (except Leflunomid) dock outside of the protein (bottom). B: This depicted conformation docks BBQ-A with the highest affinity (-12.4 kcal/mol; top, red). TCDD is docked into this conformation in a very similar pose but with lower affinity (-8.1 kcal/mol; top, green). Moreover, all 13 molecules tested were able to dock inside this conformation (bottom). The differences among these conformation sets were analyzed, compared and shown in Figures S9 and S10.

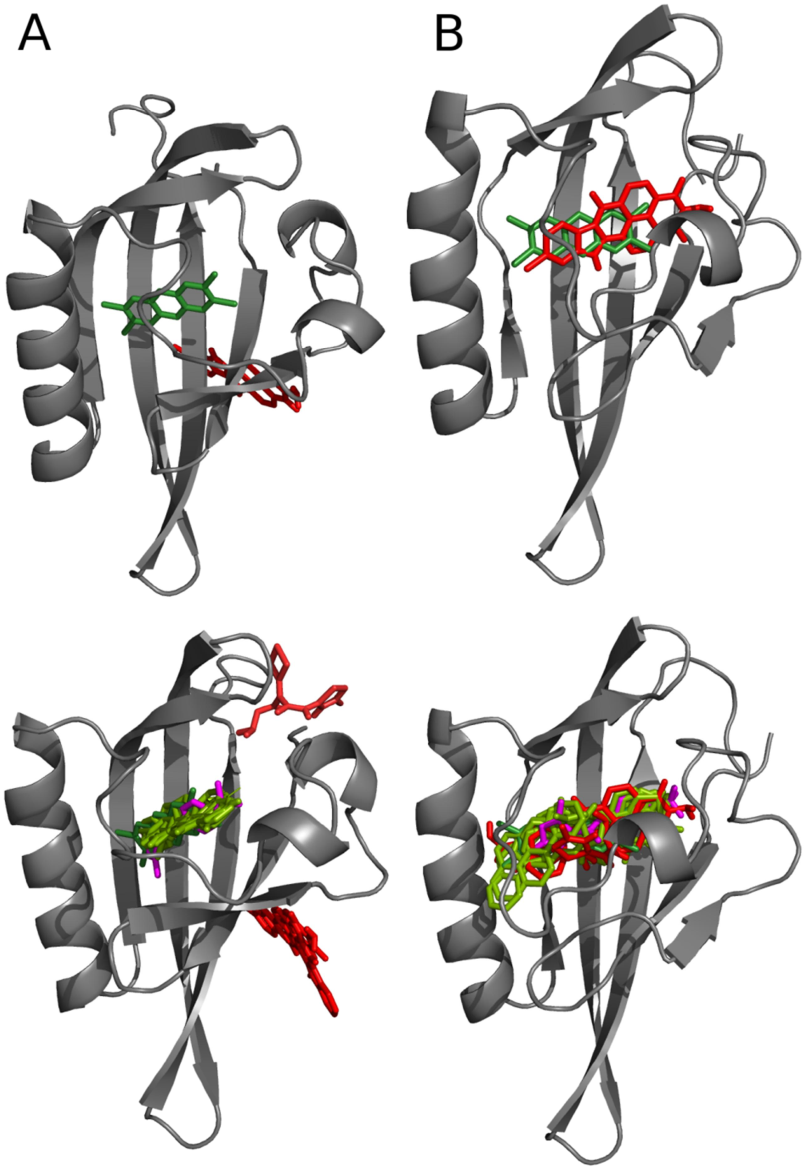

Supplement: S8 Fig — (PDF) [file pone.0146066.s008.pdf]
