# Supplementary material for: Access Path to the Ligand Binding Pocket May Play a Role in Xenobiotics Selection by AhR
Source: PLoS One. 2016 Jan 4;11(1):e0146066. doi: 10.1371/journal.pone.0146066 (PMC4699818; doi:10.1371/journal.pone.0146066)

S9 Fig. Protrusion of the amino acids delineating the pocket modulates ligand binding. The role of residues facing the cavity in binding was studied by analyzing the distance of the last side chain heavy atom of these residues from the geometric centrum of the whole domain in two sets of conformations from AhR_{CLOCK} MD CHARMM36 simulations: (1) those that bind at least one of the tested molecules (blue curves), (2) those that do not bind any of the ligands inside the pocket (red curves). TCDD fingerprint residues are labeled by asterisks. Density curves show that mostly THR289, CYS333, HIS337, MET340, SER365, ALA367, VAL381 and GLN383 can modulate ligand docking by protruding into the pocket and excluding ligands. The multimodal curves (e.g. that of PHE295, CYS333, and MET348) indicated that there are multiple stable conformations for the given residue. Moreover, these curves also reveal that some of the residue conformations are more typical for a non-binding conformation (e.g. MET340 red peak at ~8.5 Å), but are also present in the binding conformation (MET340 blue peak at ~8.2 Å). This shows that none of the tested residues alone can diminish ligand binding thus no characteristic pattern of protruding residues can be defined for binding or non-binding conformations. In other words, binding is affected by the protrusion of random sets of these residues.

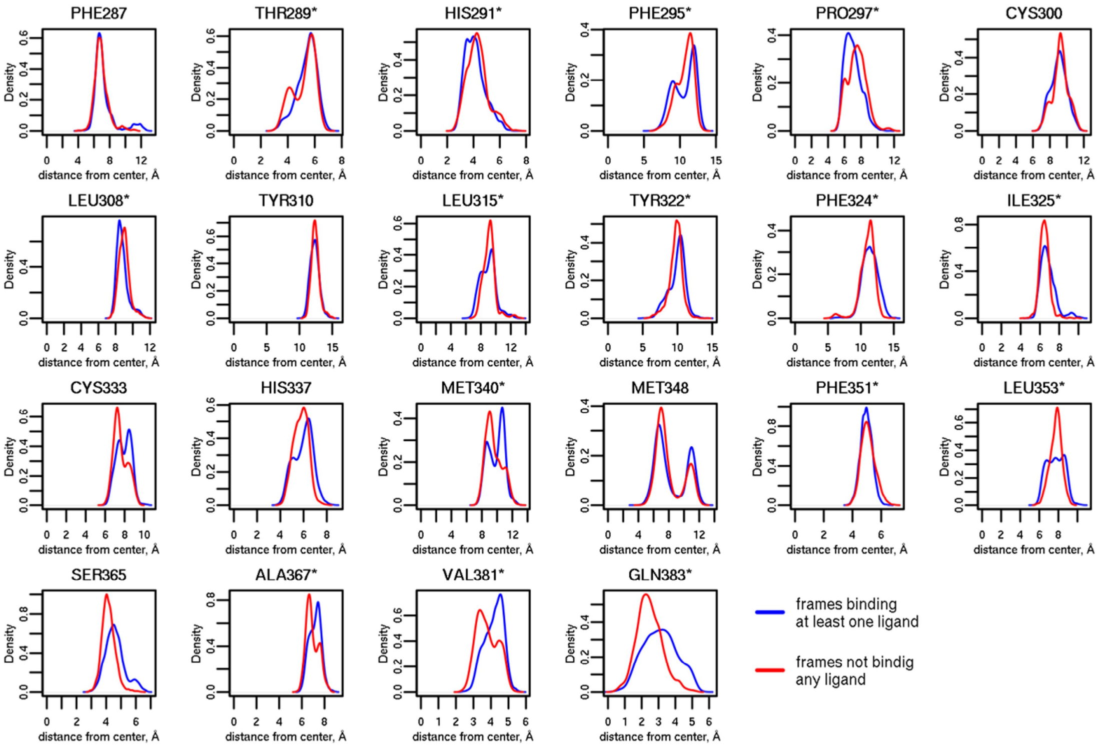

Supplement: S9 Fig — (PDF) [file pone.0146066.s009.pdf]
