# Supplementary material for: Access Path to the Ligand Binding Pocket May Play a Role in Xenobiotics Selection by AhR
Source: PLoS One. 2016 Jan 4;11(1):e0146066. doi: 10.1371/journal.pone.0146066 (PMC4699818; doi:10.1371/journal.pone.0146066)

S10 Fig. TYR322 and HIS337 may play a role in differentiating molecules with high and low affinities. The role of residues facing the cavity in binding was also studied similarly as in Fig. S9, but the conformations from AhR_{CLOCK} MD CHARMM36 simulations are grouped differently into two sets: (1) those that bind only molecules with high affinity (red curves), (2) those that bind any of the ligands (blue curves). TCDD fingerprint residues are labeled by asterisks. Conclusion very similar that in Fig. S9 can be drawn from the density curves, expect that TYR322 and HIS337 exhibit such peaks computed from frames binding only high affinity ligands (red curves), which peaks show minimal overlap with the density curves calculated from frames binding also molecules with low affinity. This observation indicates that conformation of TYR322 and HIS337 may play a more important role in discriminating high and low affinity ligands than other TCDD residues.

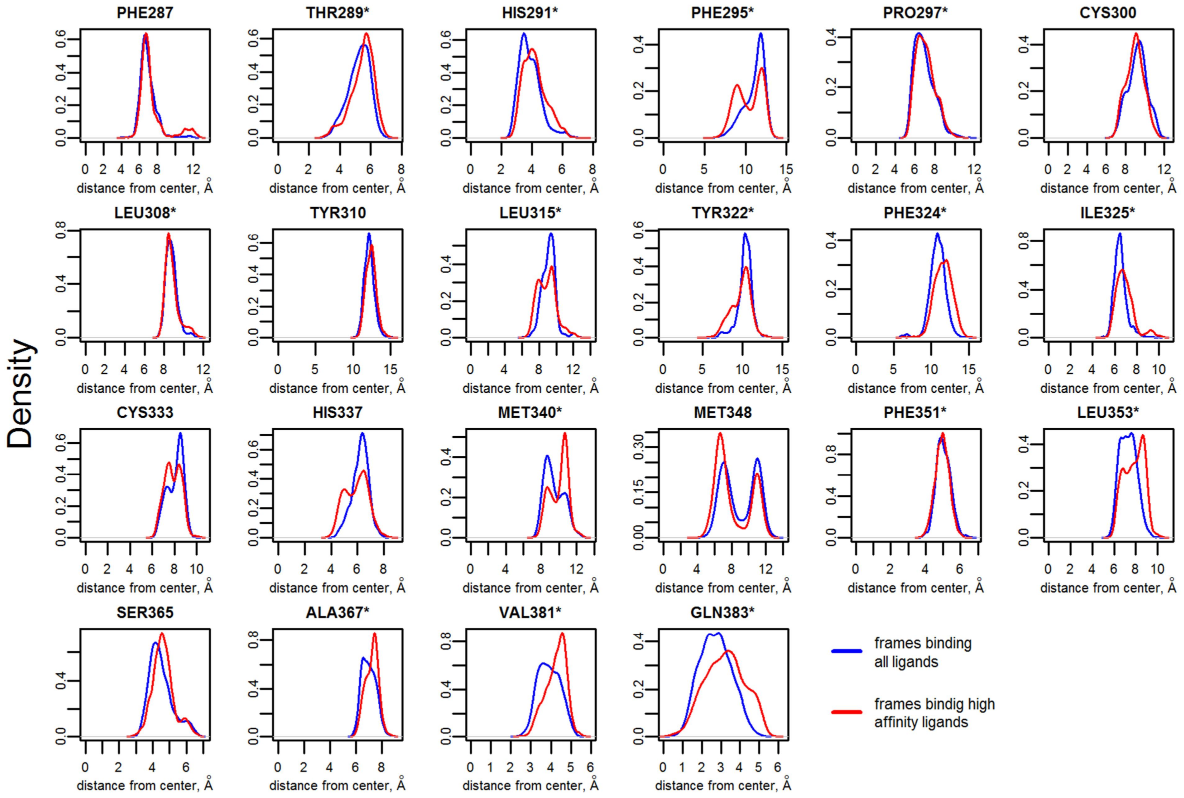

Supplement: S10 Fig — (PDF) [file pone.0146066.s010.pdf]
