# Supplementary material for: Access Path to the Ligand Binding Pocket May Play a Role in Xenobiotics Selection by AhR
Source: PLoS One. 2016 Jan 4;11(1):e0146066. doi: 10.1371/journal.pone.0146066 (PMC4699818; doi:10.1371/journal.pone.0146066)

S11 Fig. Induced Fit calculations cannot differentiate between molecules with high and low affinities. In order to incorporate structural flexibility of the binding site, Induced Fit Docking calculations were performed using the Schrödinger Suite 2015-3 software package [5,6]. After the initial docking step to the energy minimized homology models, softened potentials in Glide were employed. This allows the side-chains of the binding site residues to move and adapt to the ligand structure. After minimization of the complex structure a final docking calculation is performed. This procedure applies only molecular mechanics (MM) calculations, so it is limited to the exploration of binding site conformations. We applied the extended sampling protocol, thus 80 conformations are used for the final calculations. The plots of the results show that these MM calculations also cannot discriminate molecules with high and low affinities. The number of resulted poses is indicated in parenthesis. Green symbols indicate molecules with high affinity, while the other colors depict low affinity ligands or non-binders.

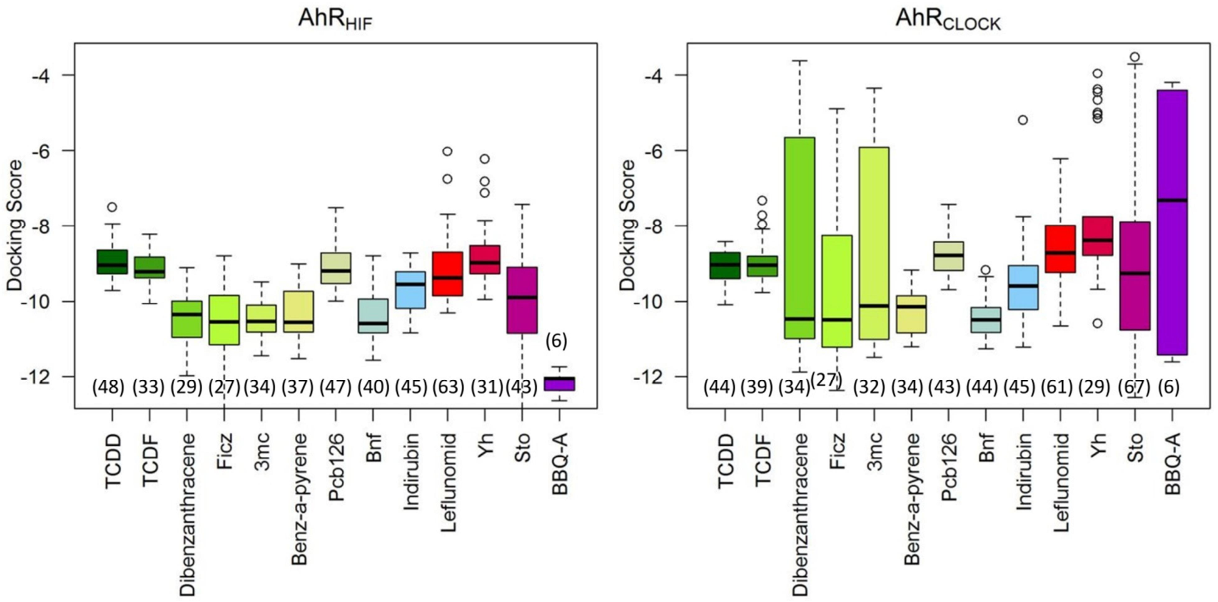

Supplement: S11 Fig — (PDF) [file pone.0146066.s011.pdf]
