# Supplementary material for: Access Path to the Ligand Binding Pocket May Play a Role in Xenobiotics Selection by AhR
Source: PLoS One. 2016 Jan 4;11(1):e0146066. doi: 10.1371/journal.pone.0146066 (PMC4699818; doi:10.1371/journal.pone.0146066)
Supplement: S12 Fig — (PDF) [file pone.0146066.s012.pdf]

S12 Fig. Complexes with high and low affinity molecules behave similarly. We aimed to perform MD and DMD simulations with the holo AhR PAS-B domain to assess whether ligands with high affinity can be discriminated from molecules with low affinity based on their effect on PAS domain dynamics. The protein conformation at 2 ns was selected from each trajectory, since all of them could dock all the ligands in a relevant pose (in the binding pocket). *In silico* docking was performed using Autodock Vina as described in the main text. The complex with the pose exhibiting the lowest binding energy for each small molecule was selected for simulations. The analysis for the AhR_{CLOCK} complexes is shown below as an example.

A: All bound molecules decrease the flexibility mostly around the N-terminal part of the belt region and the connector helix. Short (40,000 time units) DMD simulations at 0.59 temperature units with AhR PAS-B apo and complexes of TCDD, TCDF, Ficz, Bnf, BBQ-A, and Yh were performed. 100-100 parallel simulations were made to enhance the statistical relevance of the results. The short simulation time was used because ligands dismount from the PAS domain relatively fast. The mean dismounting time was 32,753 time units and no difference could be observed for molecules with different affinities (not shown). RMSF values were calculated and plotted from the part of the trajectories where the small molecule was still engaged with the domain (colored areas show ± standard deviation of the corresponding curve; n=100). Our results indicate that ligands in the complex decrease flexibility of the PAS domain independently of their affinity.

B-E: Molecules exhibit slightly different effects on PAS-B dynamics in conventional MD simulations. The same AhR PAS-B complexes of TCDD, Ficz, Bnf, and BBQ-A were also subjected to 50 ns long simulations (n=3 for each complex) using GROMACS with CHARMM36 force field complemented with parameters for small organic molecules. Complexes were prepared and the force field was generated using the CGenFF program and scripts (https://cgenff.paramchem.org) [8,9]. In these conventional MD simulations no dismounting occurred. RMSF values of the complexes showed no relevant differences (not shown). We also aimed to compare the effects of ligand binding on the residue interaction network. MDAnalysis toolkit [10] and its elastic network analysis module [11] were used similarly as for comparing the homology models (Fig. 2), but the inputs were not single structures, but full trajectories. The edges are weighted according to the presence of the corresponding interactions during simulations. Blue and red edges represent interactions characteristic for the complex and apo conformations, respectively. The effects of molecules with different activity on PAS-B domain dynamics are minimal and not consistent at these times scales. This observation may originate from insufficient sampling by atomistic MD simulations.

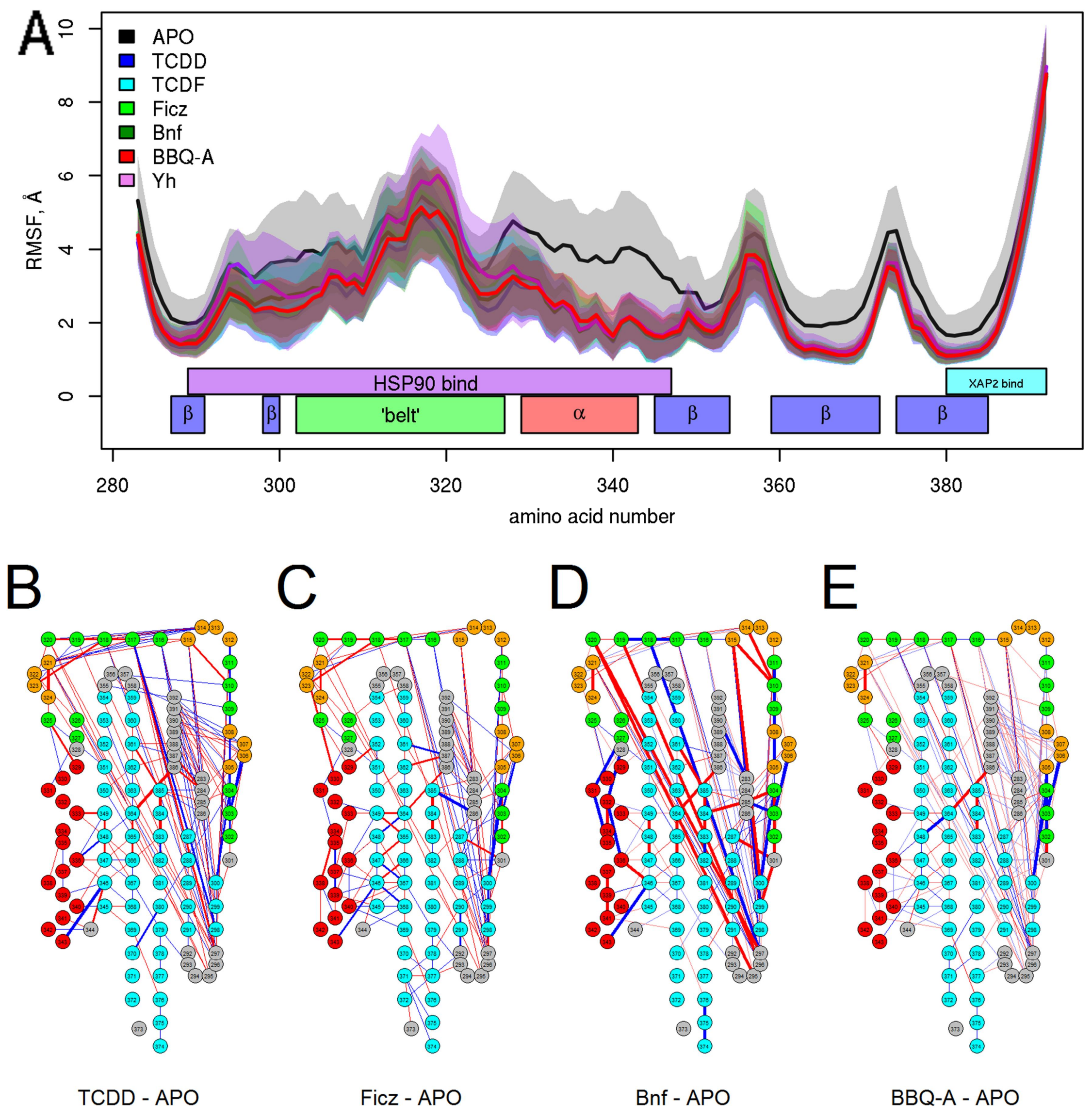
A
APO
TCDD
TCDF
Ficz
Bnf
BBQ-A
Yh
RMSF, Å
0
2
4
6
8
10
HSP90 bind
XAP2 bind
β
β
'belt'
α
β
β
β
280
300
320
340
360
380
amino acid number
B
C
D
E
TCDD - APO
Ficz - APO
Bnf - APO
BBQ-A - APO
